# Tinnitus and distress: an electroencephalography classification study

**DOI:** 10.1093/braincomms/fcad018

**Published:** 2023-02-01

**Authors:** Andrea Piarulli, Sven Vanneste, Idan Efim Nemirovsky, Sivayini Kandeepan, Audrey Maudoux, Angelo Gemignani, Dirk De Ridder, Andrea Soddu

**Affiliations:** Department of Surgical, Medical and Molecular Pathology and Critical Care Medicine, University of Pisa, Pisa 56124, Italy; Trinity College Institute for Neuroscience & School of Psychology, Trinity College, Dublin D02 PN40, Ireland; Brain Research Center for Innovative and Interdisciplinary Neuromodulation and Department of Neurosurgery, University Hospital, Antwerp 2650, Belgium; Western Institute for Neuroscience, Physics & Astronomy Department, University of Western Ontario, London, ON N6A 3K7, Canada; Department of Physics, University of Sri Jayewardenepura, Nugegoda 10250, Sri Lanka; Robert Debré University Hospital, APHP, Paris 75019, France; Department of Surgical, Medical and Molecular Pathology and Critical Care Medicine, University of Pisa, Pisa 56124, Italy; Brain Research Center for Innovative and Interdisciplinary Neuromodulation and Department of Neurosurgery, University Hospital, Antwerp 2650, Belgium; Unit of Neurosurgery, Department of Surgical Sciences, University of Otago, Dunedin, Otago 9016, New Zealand; Western Institute for Neuroscience, Physics & Astronomy Department, University of Western Ontario, London, ON N6A 3K7, Canada

**Keywords:** tinnitus, suffering, distress, machine learning

## Abstract

There exist no objective markers for tinnitus or tinnitus disorders, which complicates diagnosis and treatments. The combination of EEG with sophisticated classification procedures may reveal biomarkers that can identify tinnitus and accurately differentiate different levels of distress experienced by patients. EEG recordings were obtained from 129 tinnitus patients and 142 healthy controls. Linear support vector machines were used to develop two classifiers: the first differentiated tinnitus patients from controls, while the second differentiated tinnitus patients with low and high distress levels. The classifier for healthy controls and tinnitus patients performed with an average accuracy of 96 and 94% for the training and test sets, respectively. For the distress classifier, these average accuracies were 89 and 84%. Minimal overlap was observed between the features of the two classifiers. EEG-derived features made it possible to accurately differentiate healthy controls and tinnitus patients as well as low and high distress tinnitus patients. The minimal overlap between the features of the two classifiers indicates that the source of distress in tinnitus, which could also be involved in distress related to other conditions, stems from different neuronal mechanisms compared to those causing the tinnitus pathology itself.

## Teaser

In this study, Piarulli *et al*. conduct a thorough classification procedure for EEG data acquired from tinnitus patients and healthy controls. The authors develop two classifiers that can accurately distinguish tinnitus patients from healthy controls as well as tinnitus patients with low and high distress levels.

## Graphical Abstract

**Graphical abstract:**
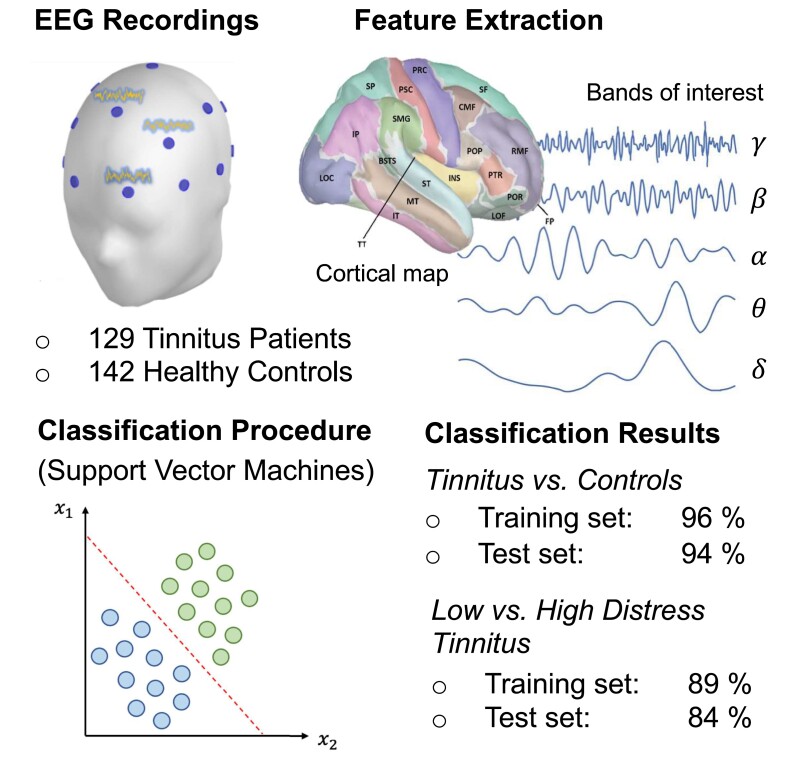

## Introduction

In recent years, many efforts have been made to implement sophisticated classification procedures in conjuncture with neuroimaging and electrophysiological techniques.^1-3^ However, more work is needed to implement such methods in the assessment and diagnosis of neurophysiological pathologies and mental health disorders. One condition on which we can greatly improve our understanding is tinnitus. Tinnitus can be defined as the conscious awareness of noise or tone for which there is no corresponding external acoustic source, and individuals are diagnosed with a tinnitus disorder if this awareness results in distress.^4-6^ In other words, tinnitus itself describes an auditory phantom sound, whereas any associated suffering constitutes a tinnitus disorder. Unfortunately, tinnitus is a widespread pathology that affects up to 25.3% of the American population.^7,8^ The most common form is subjective tinnitus, which arises from neuronal pathologies and is therefore primarily assessed by the self-report of patients.^9^ Clinicians are not able to identify or measure subjective tinnitus, and consequently, an accurate means for identifying the presence of this condition and the level of distress it induces is lacking. Moreover, treatments for subjective tinnitus tend to focus on reducing psychological side effects rather than addressing the physiological causes of the phantom noise.^10,11^

Given these challenges, this study aims to identify objective electrophysiological markers that are associated with tinnitus and the level of distress experienced by patients. To do so, we conducted cortical EEG measurements on a group of tinnitus patients and healthy controls. The popularity of EEG continues to grow due to its low cost compared to neuroimaging tools (i.e. fMRI and PET) and its relative ease of employment in clinical and research settings. Early studies that used EEG to compare tinnitus patients and healthy controls focused on specific electrical features that differ between the two groups, such as event-related potentials and power over frequency bands.^12,13^ More recent works have implemented machine learning to study tinnitus with EEG and other tools such as near-infrared spectroscopy.^14,15^ The findings of these studies indicate the potential to differentiate tinnitus patients from healthy controls based on connectivity features and sample entropy.^15,16^ These preliminary results are promising, and our study expands on them by implementing intensive classification procedures that use a wider range of candidate features and sizable samples of subjects. We also develop a classifier specific to tinnitus-induced distress, which we use to further classify tinnitus patients as having high or low distress levels. More specifically, our features are derived using a cortical source reconstruction and parcellation scheme. Beyond pointing out the signal characteristics associated with tinnitus, we expect that this approach will allow us to localize these features to specific cortical areas. Finally, we interpret these findings in the context of existing literature on tinnitus and discuss how our implementation of machine learning can advance diagnosis and treatments for this condition.

## Materials and methods

### Participants

One hundred and twenty-nine tinnitus patients (38 females, mean age = 50 and SD = 15) and 142 healthy controls (52 females, mean age = 47 and SD = 17) participated in this study. The controls were selected from a convenience sample of 269 healthy individuals to obtain a group comparable to the patients in terms of numerosity, age (*t* = 1.20, *P* < 0.23 and unpaired *t*-test) and gender composition (χ^2^ = 1.26, *P* < 0.27, χ^2^ test with Yates’s correction).

Tinnitus patients were evaluated for distress using the tinnitus questionnaire (TQ), which consists of questions about phantom noise and how it affects their physical and mental well-being.^17^ Hearing levels were assessed using classical audiological testing, in which pure tones ranging from 250 Hz to 8 kHz were presented to each ear until the threshold of detection was reached. Following the World Health Organization guideline for grading hearing impairment, the mean audiometric threshold was calculated by averaging the hearing thresholds at 500, 1000, 2000 and 4000 Hz.^18^

Demographic and behavioural features of tinnitus patients, as well as TQ scores and hearing levels, are presented in Supplementary Appendix A. The study was approved by the local ethical committee (Antwerp University Hospital) and was in accordance with the declaration of Helsinki. All participants provided written informed consent.

### Study design

#### EEG pre-processing

Each subject underwent a resting-state EEG recording with their eyes closed for a duration of 5 min. EEG was performed using Mitsar-201 amplifiers (NovaTech EEG: www.novatecheeg.com/), with 19 sensors positioned according to the 10–20 International System. The sampling rate was 128 Hz, and a 2–44 Hz bandpass filter was applied.

The artefact cleaning procedure comprised of the following: (i) independent component analysis^19^ to identify and remove stationary artefacts such as eye-blinking and (ii) a visual inspection of the epochs to remove contamination by other movement artefacts not identified with independent component analysis. Channels were then re-referenced using the average signal.

#### Cortical source reconstruction

For cortical source reconstruction, a forward model was generated using a symmetric boundary element method (OpenMEEG software)^20^ based on the MNI152 standard head-model. The time courses of standardized cortical current densities (sLORETA)^21^ were estimated for 15 000 voxels using toolbox functions in Brainstorm.^22^

**Figure 1:**
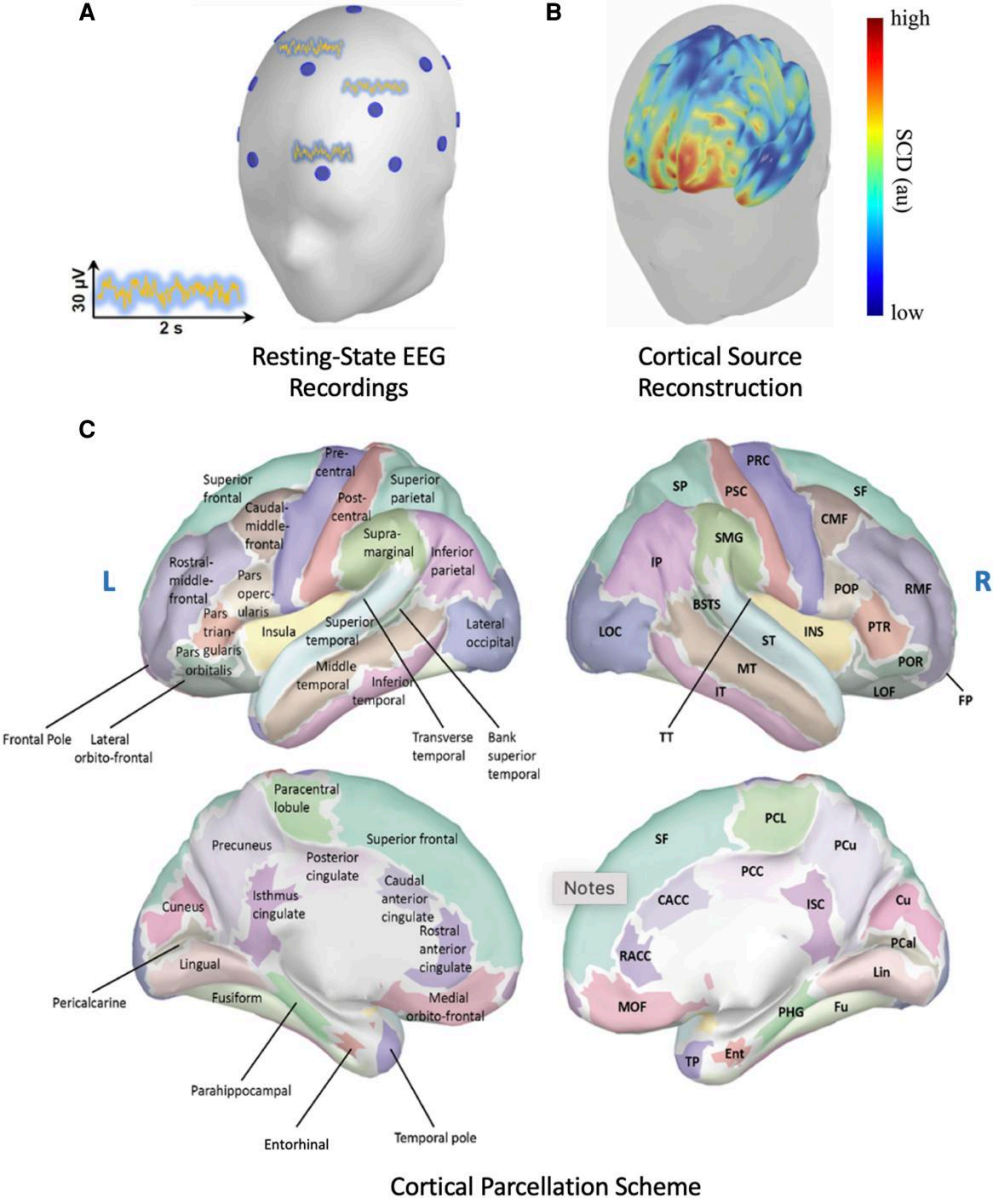
**Summary of methods and parcellation scheme.** From EEG measurements to the parcellation scheme: (**A**) resting-state EEG was recorded at 19 positions following the 10–20 International System. (**B**) Estimation of standardized cortical current densities using sLORETA (au stands for arbitrary units). (**C**) The Desikan-Killiany parcellation scheme: on the left hemisphere, cortical areas are presented with their full names, whereas on the right hemisphere, cortical areas are identified with the following abbreviations: BSTS: bank of the superior temporal sulcus; CACC: caudal anterior cingulate cortex; CMF: caudal middle frontal cortex; Cu: cuneus; Ent: entorhinal cortex; FP: frontal pole; Fu: fusiform gyrus; IP: inferior parietal cortex; IT: inferior temporal cortex; ISC: isthmus of the cingulate cortex; LOC: lateral occipital cortex; LOF: lateral orbito-frontal cortex; Lin: lingual gyrus; MOF: medial orbito-frontal cortex; MT: middle temporal cortex; PCL: paracentral lobule; PHG: parahippocampal gyrus; POP: pars opercularis; POR: pars orbitalis; PTR: pars triangularis; PCal: pericalcarine cortex; PSC: postcentral gyrus; PCC: posterior cingulate cortex; PRC: precentral gyrus; PCu: precuneus; RAC: rostral anterior cingulate cortex; RMF: rostral middle frontal cortex; SF: superior frontal cortex; SP: superior parietal cortex; ST: superior temporal cortex; SMG: supramarginal Gyrus; TP: temporal pole; TT: transverse temporal cortex, INS: Insula.

The cortex was segmented using the Desikan-Killiany atlas^23^ and time courses were assigned to each of the 68 regions by averaging the time courses of their constituent voxels. The obtained signals were then band-pass filtered (Chebishev type II filters) to obtain time courses for six frequency bands of interest: *δ* (2–3.75 Hz); *θ* (4–7.75 Hz); *α* (8–11.75 Hz); *β*_1_ (12–17.75 Hz); *β*_2_ (18–29.75 Hz) and *γ* (30–44 Hz). The acquisition procedure and the 68 regions obtained with it are summarized in Fig. 1.

#### Cortical power spectral densities

For each region, time courses were divided into 4 s epochs (50% overlap between contiguous epochs). For each epoch, the power spectral density (PSD) was estimated by applying a Hamming-windowed fast Fourier transform, which was then log-transformed. The mean power of each frequency band was estimated for each epoch by averaging over the band’s frequency bins. Then, each band’s average PSD for each region was obtained by averaging over the epochs. For relative PSDs, we computed the ratio between the power in a specific band and the total power in the entire 2–44 Hz domain.

#### Lempel–Ziv complexity

The next feature included was Lempel–Ziv complexity (LZC),^24^ which is a metric that quantifies a signal’s complexity based on the number of bits required to generate it. LCZ was estimated for all bands of interest and cortical regions on 20-s consecutive epochs. Within the bands, current density signals were first converted into binary sequences using the binarization threshold introduced by Schartner *et al*.^25^ The complexity for each subject, band and the region was then estimated by averaging over the epochs.^26^

#### Functional connectivity

The filtered signals were segmented into 4-s contiguous epochs. For each band and epoch, phase transfer entropy (PTE) was calculated between each pair of regions. PTE is a directional measure of connectivity used for a given pair of regions (i.e. X and Y). Based on the direction of the information flow (IF), two values are extracted (X→Y and Y→X).^27^ When PTE was computed, the average connectivity map was obtained for each subject and band by averaging connectivity measurements over epochs.

#### Information flow

We then estimated the total information flowing into and out of each region. Considering any region *i* from those obtained with the Desikan-Killiany atlas, the outward IF (IF_*O*_) and its inward counterpart (IF_*I*_) were computed as follows: $IF_{O}=\overset{n}{\underset{\;j=1}{\sum }}\;\mathrm{PT}E_{i\to j}\;,\;j\neq i$ and $IF_{I}=\overset{n}{\underset{\;j=1}{\sum }}\;\mathrm{PT}E_{\;j\to i}\;,\;j\neq i$. The IF between two regions is a type of functional connectivity measure, and with direction considered, reflects the influence of one region over the other through information transfer.^28^ By summing over all a region’s inward and outward values as done for IF_*O*_ and IF_*I*_, we obtain a cumulative measure of the region’s dynamic interactions with all other regions.

#### Graph theory metrics

Each band’s bidirectional PTE values were obtained for every pair of electrodes and were used to construct non-symmetric 68 × 68 connectivity matrices. A range of thresholds was applied by retaining between 90 and 10% of the higher connectivity values in steps of 2.5%.^29,30^ For each iteration, the following graph-theoretical parameters were obtained by using the brain connectivity toolbox^31^: clustering coefficient, global efficiency, graph strength, modularity, in-degree participation coefficient and out-degree participation coefficient. For each subject and band, these metrics were averaged over the range of thresholds applied.

#### Statistical analysis

When appropriate, between-group comparisons were conducted using unpaired *t*-tests (controls versus tinnitus patients and high versus low distress). Comparisons on binary variables (e.g. gender, tinnitus type and side) were instead performed using *χ*^2^ tests with Yates correction.^32^ Threshold for significance was set at *P* = 0.05.

### Classification procedures

For the tinnitus versus controls (C–T) classification procedure, we used measurements from the 142 healthy controls and the 129 tinnitus patients. In the low versus high distress (L–H) classification, the patients were divided into two non-overlapping groups based on their TQ distress scores. The low distress group (51 patients) included subjects with a TQ score < 30, while the high distress group included those with a TQ score > 38. Patients with TQ scores near the median of the distribution (22 patients, median score of 34), were excluded from this classification, leaving a total of 107 subjects (see Supplementary Appendix A for the demographic and behavioural details of the two groups).

For each subject, the features described in this section were collected for each of the six bands of interest: (i) absolute PSDs; (ii) relative PSDs; (iii) LZC; (iv) inward flow of information; (v) outward flow of information and (vi) graph theory metrics (clustering coefficient, global efficiency, graph strength, modularity, in-degree participation coefficient and out-degree participation coefficient). Note that features 1–5 were applied to each individual region of the Desikan-Killiany atlas, while graph theory measures were global parameters.

#### Step 1: *P*-value comparison

First, the electrophysiological features were used to conduct between-group comparisons for both C–T and L–H classifications. Only features showing significant between-group differences were retained for further analyses, with significance set to *P* < 0.05 without correction for multiple comparisons.

#### Step 2: feature selection algorithm

The remaining features were standardized using a *z*-score transformation. We then selected the subset of features with the highest performance in discriminating between the two groups as follows: each dataset was submitted to a feature selection algorithm that uses neighbourhood component analysis,^33^ an algorithm that searches for the features that maximize a classifier’s prediction accuracy. This was run 1000 times; at each step, we collected the weight of each feature, which reflects its contribution to the between-group discrimination. Only features with higher discriminatory capacity were kept for further analysis. The efficacy of the selected features was then verified using support vector machines (SVMs),^34^ a common classification algorithm. We ran SVMs with 5-folds cross-validation 1000 times to ensure stable validation accuracy.

#### Step 3: optimization of feature selection

Having proven the efficacy of the selected features in both C–T and L–H classifiers, we then reduced the number of features to optimize each classifier’s performance: a step-down procedure was used to eliminate redundant parameters while monitoring validation accuracy levels.

#### Step 4: classifier training and testing

With the number of features optimized, the classifiers were tested and validated using a hold-out procedure. For both C–T and L–H classifiers, 70% of subjects were put into the training set, the part of the data used to optimize the classifier’s parameters, and 30% were put in the test set, the remaining sample used to evaluate the classifier’s performance on new data (C–T training: 99 controls and 90 patients, C–T test: 43 controls and 39 patients, L–H training: 39 low distress and 35 high distress and L–H test: 16 low distress and 17 high distress). As the choice of subjects in the training and test sets was arbitrary, we trained both classifiers 5000 times with random assignments of subjects to the two sets.

## Results

### Controls–tinnitus classification

#### Steps 1–3: features/parameters selection

At the end of the first selection step, which was based on between-group statistical differences, 1384 candidate features were retained. The second step of feature selection resulted in a total of 58 features. Finally, this number was reduced to 23 features after the optimization procedure.

#### Step 4: classifier training and testing

The average accuracies reached on the training and test sets were 96 and 94%, respectively. The true positive rate was 96.8% on the training set and 95.4% on the test set. For true negative rates, the classifier reached values of 95.2% on the training set and 94.0% on the test set.

### High–low distress classification

#### Steps 1–3: features/parameters selection

For the selection using between-group statistical differences, 414 candidate features were retained. The second step of feature selection yielded 41 features, which was reduced to 20 features in the optimization procedure. For more details on the results obtained at each step for the two classifiers, see Supplementary Appendices B and C.

#### Step 4: classifier training and testing

The distress classification had an average accuracy of 89% for the training set and 84% for the test set. The classifier had a true positive rate of 94% on the training set and a rate of 87% on the test set. For the negative rates, the values were 86% on the training and 82% on the test set.

**Figure 2:**
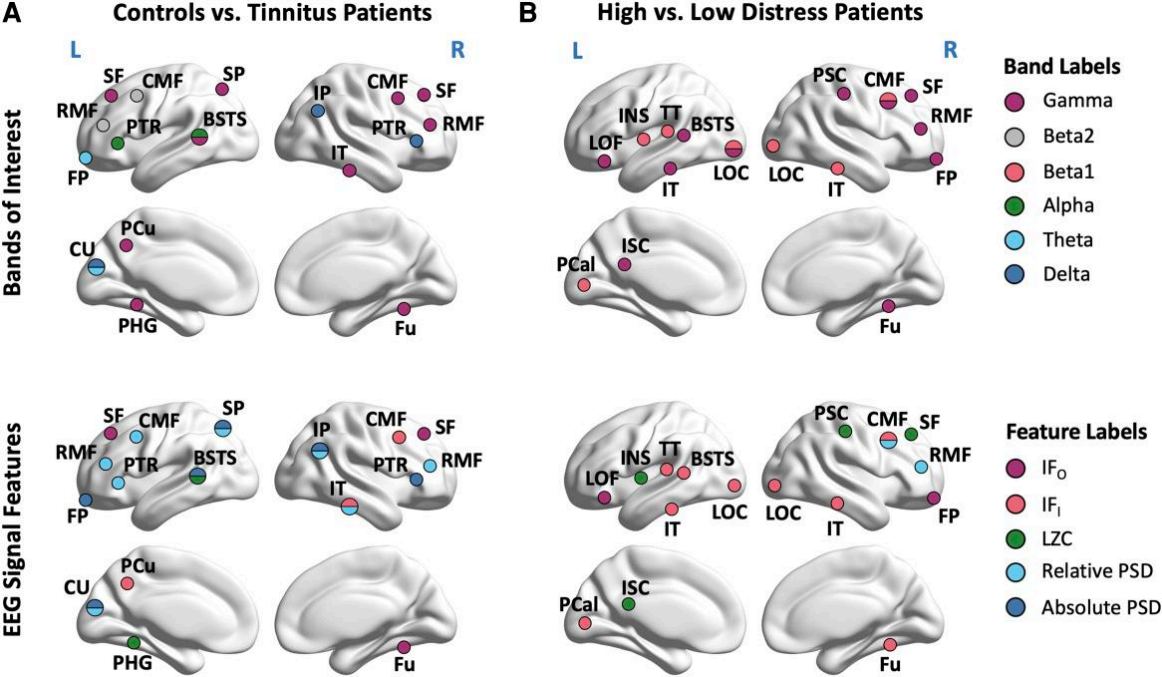
**Cortical areas and EEG features of the classifiers.** In column A, we present the cortical areas of the tinnitus patients versus controls classifier, while those involved in discriminating between tinnitus patients with low and high distress are presented in column B. Cortical areas are identified by the frequency band(s) in which EEG-derived features were observed in the first row and by the specific EEG-derived features in the second row. Note that in the low versus high tinnitus classifier, two additional EEG features (derived from graph theory analysis) were selected: gamma global efficiency and graph strength.

**Figure 3:**
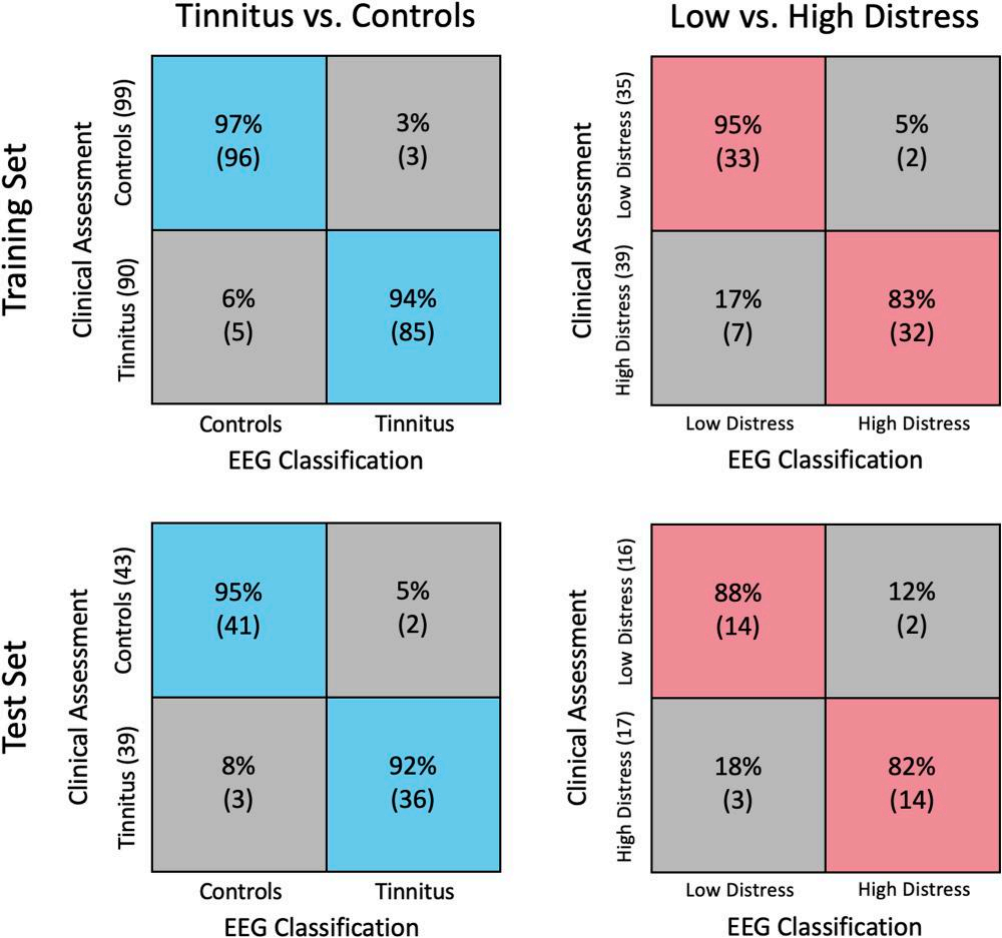
**Confusion matrices for classification results.** Confusion matrices for the tinnitus versus control classifier are presented in the first column, while those for the low versus high tinnitus distress classification are presented in the second column. The performances on the training and test sets are presented in the first and second rows, respectively, and are based on 5000 runs of the classifier (see main text). On each confusion matrix, the clinical assessment outcomes are shown on the *y*-axis (with the total number of subjects written in brackets), while the predictions based on EEG features are shown on the *x*-axis. The diagonal elements of each matrix identify the percentages of correct classifications (true positive and negative rates) for each category (the corresponding number of subjects is reported between brackets), while the off-diagonal elements identify the percentages of misclassified subjects (false positives and negatives). For each entry, the corresponding number of subjects that fell into the classification is shown in brackets.

In Fig. 2, each classifier’s features are shown using a visuospatial representation, highlighting the cortical areas corresponding to each feature. These cortical areas of selected features are also presented in Table 1, where regions are grouped based on frequency bands (rows) and feature types (columns). Finally, we show each classifier’s confusion matrices in Fig. 3 for the training and test sets.

**Table 1:** The EEG-derived features of both classifiers are organized in this table according to their type (columns) and frequency band (rows)

	Absolute PSD	Relative PSD	Complexity	Inward flow	Outward flow
*γ*	**BSTS_L_ | SF_R_ | SP_L_**	**IT_R_ | SP_L_** CMF_R_ | RMF_R_	**PHG_L_** ISC_L_ | PSC_R_	**CMF_R_ | IT_R_ | PCU_L_** BSTS_L_ | Fu_R_ | IT_L_ | LOC_R_	**Fu_R_ | SF_L_** FP_R_ | LOF_L_
*β*_2_	**FP_L_**	**CMF_L_ | RMF_L_**	—	—	—
*β*_1_	—	—	INS_L_ | SF_R_	CMF_L_ | ITR | LOC_L_ LOC_R_ | PCal_L_ | TTL	—
*α*	—	PTR_L_	**BSTS_L_**	—	—
*θ*	**Cu_L_**	—	—	—	—
*δ*	**Cu_L_ | IP_R_ | PTR_R_**	**Cu_L_ | IP_R_ | RMF_R_**	—	—	—

The entries are filled with the region abbreviations to which the features corresponded, with bolded text for the C–T classifier and regular text for the L–H classifier. No graph theory metrics were observed for the C–T classifier, whereas the L–H classifier consisted of seven. This table and Fig. 2 also emphasize the minimal overlap in regions and features between the two classifiers. BSTS: bank of the superior temporal sulcus; CACC: caudal anterior cingulate cortex; CMF: caudal middle frontal cortex; Cu: cuneus; Ent: entorhinal cortex; FP: frontal pole; Fu: fusiform gyrus; IP: inferior parietal cortex; IT: inferior temporal cortex; ISC: isthmus of the cingulate cortex; LOC: lateral occipital cortex; LOF: lateral orbito-frontal cortex; Lin: lingual gyrus; MOF: medial orbito-frontal cortex; MT: middle temporal cortex; PCL: paracentral lobule; PHG: parahippocampal gyrus; POP: pars opercularis; POR: pars orbitalis; PTR: pars triangularis; PCal: pericalcarine cortex; PSC: postcentral gyrus; PCC: posterior cingulate cortex; PRC: precentral gyrus; PCu: precuneus; RAC: rostral anterior cingulate cortex; RMF: rostral middle frontal cortex; SF: superior frontal cortex; SP: superior parietal cortex; ST: superior temporal cortex; SMG: supramarginal gyrus; TP: temporal pole; TT: transverse temporal cortex, INS: Insula.

## Discussion

The C–T classifier consisted of 23 EEG features including the following: absolute PSD in the *δ* and θ bands, as well as in the higher *β*_2_ and *γ* frequencies; relative PSD, which was also in both low and high frequencies but with *α* instead of *θ*; signal complexity in the *α* and *γ* bands; inward and outward information over five regions and only in the *γ* band.

On the other hand, the 20 features of the tinnitus distress (L–H) classifier were predominantly in the *β*_1_ and *γ* frequency bands. This is in accordance with existing literature that associates external and phantom auditory perception with high-frequency band activities.^35-38^ Only two features were associated with PSD, both of which were relative and in the *γ* band. Moreover, four complexity features were extracted, two in each of the *β*_1_ and *γ* bands. The largest number of features were related to IF, with 10 inward values shared between the same two frequencies and two outward values for the *γ* band. Finally, seven graph theory metrics were extracted to differentiate low and high distress, all associated with the *γ* band.

Comparing the two classifiers, no overlapping bands or regions were observed for PSD and complexity. The only feature with overlapping regions was inward IF [right caudal middle frontal (CMF) and the right inferior temporal (IF) cortices], but with differing frequencies for each classifier (*β*_1_ for L–H and *γ* for C–T).

Other than frequency bands, the types of features varied significantly between the two classifiers. The tinnitus classifier had significantly more PSD features, while the distress classifier was the only one with graph theory metrics. Moreover, the latter also had more features derived from IF, a measure of functional connectivity between different cortical regions.^28^ These differences may suggest that the presence of tinnitus primarily depends on power-based properties in individual brain regions. On the other hand, distress and its perception likely arise from global aspects of brain connectivity, which are reflected by the graph theory and IF metrics included in the second classifier. Finally, while neither classifier had many features derived from LZC, more were present in the distress classifier, which may reflect previous findings on increased LZC in heightened states of awareness and distress.^39,40^

Once all features were obtained, we moved to training and testing. First, we achieved 96% accuracy on the training set for classifying tinnitus patients and controls, which brought us to a 94% accuracy in the test set. In the distress classifier, the training and test set accuracies were 89 and 84%, respectively.

The high accuracies of both classifiers are very promising, as they emphasize the significance of electrophysiological features as markers of tinnitus and distress. In Supplementary Appendix C, we investigated the effects of adding demographic and behavioural characteristics as features (i.e. age and level of hearing loss) to the distress classifier. With all these features and their possible combinations, the accuracy of the classifier was either reduced or not significantly improved, further emphasizing the reliability of using electrophysiological features to differentiate distress levels.

Since tinnitus is commonly associated with hearing loss, there was a possibility that the features we identified were influenced by a combination of both hearing loss and tinnitus. To test this, we employed the tinnitus-control classifier and tested its performance in classifying all controls and the subgroup of tinnitus patients who did not have a hearing impairment (81 patients or 61% of the patient population). The overall classification accuracy was 96.4% and only three patients that had tinnitus without a hearing impairment tinnitus were misclassified. The high accuracy obtained in this test emphasizes that the features used in the tinnitus-control classifier are predominantly related to tinnitus and are unlikely to be influenced by hearing loss (see Supplementary Appendix B for more details).

The first classifier can serve as a useful diagnostic tool, as it introduces an objective assessment of a disorder that is currently diagnosed using subjective self-report. At the same time, the ability to classify distress can serve as an important prognostic tool that can monitor treatment efficacy.

Another important finding is the limited amount of overlap between the features of the two classifiers, which supports the idea that the neuroelectric markers of distress differ from those that are associated with the phantom noise. This is in keeping with psychosurgery data from as early as the 1950s when it was found that lobotomies improved distress without reducing the perception of loudness in tinnitus.^41,42^ Moreover, EEG was previously used to detect differences in electrophysiological traits between tinnitus presence and tinnitus distress.^43^ This is also consistent with the current consensus on treatments for tinnitus, which aim at lowering distress without reducing perceived loudness.^44,45^

It is important to note that while the classifiers reached high accuracies, the off-diagonal elements in the confusion matrices (Fig. 3) indicate that misdiagnosis may occur, leading to tinnitus patients being diagnosed as controls and vice-versa. From a technical standpoint, this was a binary classification procedure based on a learned boundary separating two rigid classes, and it is possible that misdiagnosed cases landed close to the boundary generated by the SVMs. Although the accuracy of our classifiers suggests common features underlying tinnitus and distress in all patients, these are conditions with many potential aetiologies, which may also contribute to the diagnostic error we observed.

Furthermore, the generalizability of these results must be thoroughly assessed before the proposed procedure can be implemented in clinical practice. Source reconstruction, and hence the features extracted with it, have a dependence on the adopted EEG montage,^46^ which may be a factor affecting the performance of our classifiers. It is also important to note that while our approach relied on SVMs, other types of supervised classification procedures exist.^2^ Another important category includes deep learning classification procedures with neural networks,^47,48^ which may be more suitable for specific types of EEG analysis.

Moving forward, the relationship between condition presence and distress should be explored in other pathologies, such as chronic pain or post-traumatic stress disorders.^49^ Perhaps, the differences in features we report could apply to other conditions associated with distress, which may lead to valuable findings on distress as a general phenomenon. In conclusion, our findings emphasize the importance of establishing an objective detection of tinnitus and the distress it induces. We stress that to improve diagnosis, our proposed methodology is intended to complement rather than replace traditional diagnostic methods, such as the subjective self-reports of patients. Implementing objective assessments of tinnitus will be of great use to clinicians, especially in severe cases where patients may be unable to communicate their condition.

## Supplementary Material

fcad018_Supplementary_Data

## Data Availability

The data used in this study are not publicly available, although the authors are willing to share it upon reasonable request.

## Abbreviations

BSTS: bank of the superior temporal sulcus
CACC: caudal anterior cingulate cortex
CMF: caudal middle frontal cortex
C–T: controls–tinnitus classification
Cu: cuneus
Ent: entorhinal cortex
FP: frontal pole
Fu: fusiform gyrus
IF: information flow
INS: insula
IP: inferior parietal cortex
ISC: isthmus of the cingulate cortex
IT: inferior temporal cortex
LZC: Lempel-Ziv complexity
Lin: lingual gyrus
LOC: lateral occipital cortex
LOF: lateral orbito-frontal cortex
LZC: Lempel–Ziv complexity
L–H: low–high distress classification
MOF: medial orbito-frontal cortex
MT: middle temporal cortex
PCL: paracentral lobule
PCal: pericalcarine cortex
PCC: posterior cingulate cortex
PCu: precuneus
PHG: parahippocampal gyrus
POP: pars opercularis
POR: pars orbitalis
PRC: precentral gyrus
PSC: postcentral gyrus
PSD: power spectral density
PTE: Phase Transfer Entropy
PTR: pars triangularis
RAC: rostral anterior cingulate cortex
RMF: rostral middle frontal cortex
SF: superior frontal cortex
SMG: supramarginal gyrus
SP: superior parietal cortex
ST: superior temporal cortex
SVM: support vector machine
TP: temporal pole
TQ: tinnitus questionnaire
TT: transverse temporal cortex
