# Supplementary material for: Tinnitus and distress: an electroencephalography classification study
Source: Brain Commun. 2023 Feb 1;5(1):fcad018. doi: 10.1093/braincomms/fcad018 (PMC9927883; doi:10.1093/braincomms/fcad018)
Supplement: fcad018_Supplementary_Data [file fcad018_supplementary_data.docx]

**Tinnitus and Distress: An Electroencephalography Classification Study**

**Supplementary Material**

**Table of Contents**

| **Section** | **Page** |
| --- | --- |
| ***Supplementary Appendix A*** |  |
| Demographic and Behavioural Characterization of Tinnitus Patients | 2 |
| ***Supplementary Appendix B*** |  |
| Desikan-Killiany Atlas and Classification Procedure | 6 |
| Tinnitus vs. Controls Classifier Results | 9 |
| ***Supplementary Appendix C*** |  |
| High vs. Low Distress Classifier Results | 27 |
| ***References*** | 39 |

**Supplementary Appendix A**

**Demographic and Behavioral Characterization of High and Low Distress Tinnitus Patients**

| **Features** |  | **High & Low Distress**  **(HD & LD)** |
| --- | --- | --- |
|  |  |  |
|  |  |  |
| **Age** (years) |  | 49.7 ± 1.3 |
| **Gender**  (males - females) |  | 91 - 38 |
| **Hearing Loss** (dB) |  | 24.4 ± 1.5 |
| **Tinnitus Type**  (narrow band - pure tone) |  | 58 - 71 |
| **Tinnitus Side**  (unilateral - bilateral) |  | 96 -33 |
| **Tinnitus Duration** (years) |  | 5.6 ± 0.6 |
| **Tinnitus Frequency** (Hz) |  | 5166 ± 280 |
| **Hearing Loss at Tinnitus Frequency** (dB) |  | 43.5 ± 2.51 |
| **Loudness at Tinnitus Frequency** (sensation, dB) |  | 7.9 ± 0.8 |
| **Tinnitus Questionnaire: Distress** |  | 36 ± 1.5 |
| **Subjective Loudness (VAS)** |  | 5.3 ± 0.2 |

**Supplementary Table 1.** Descriptive statistics of tinnitus patients’ demographic and behavioural characteristics. Statistics are presented as mean ± standard error**,** unless exact numbers of patients are reported for a certain feature.


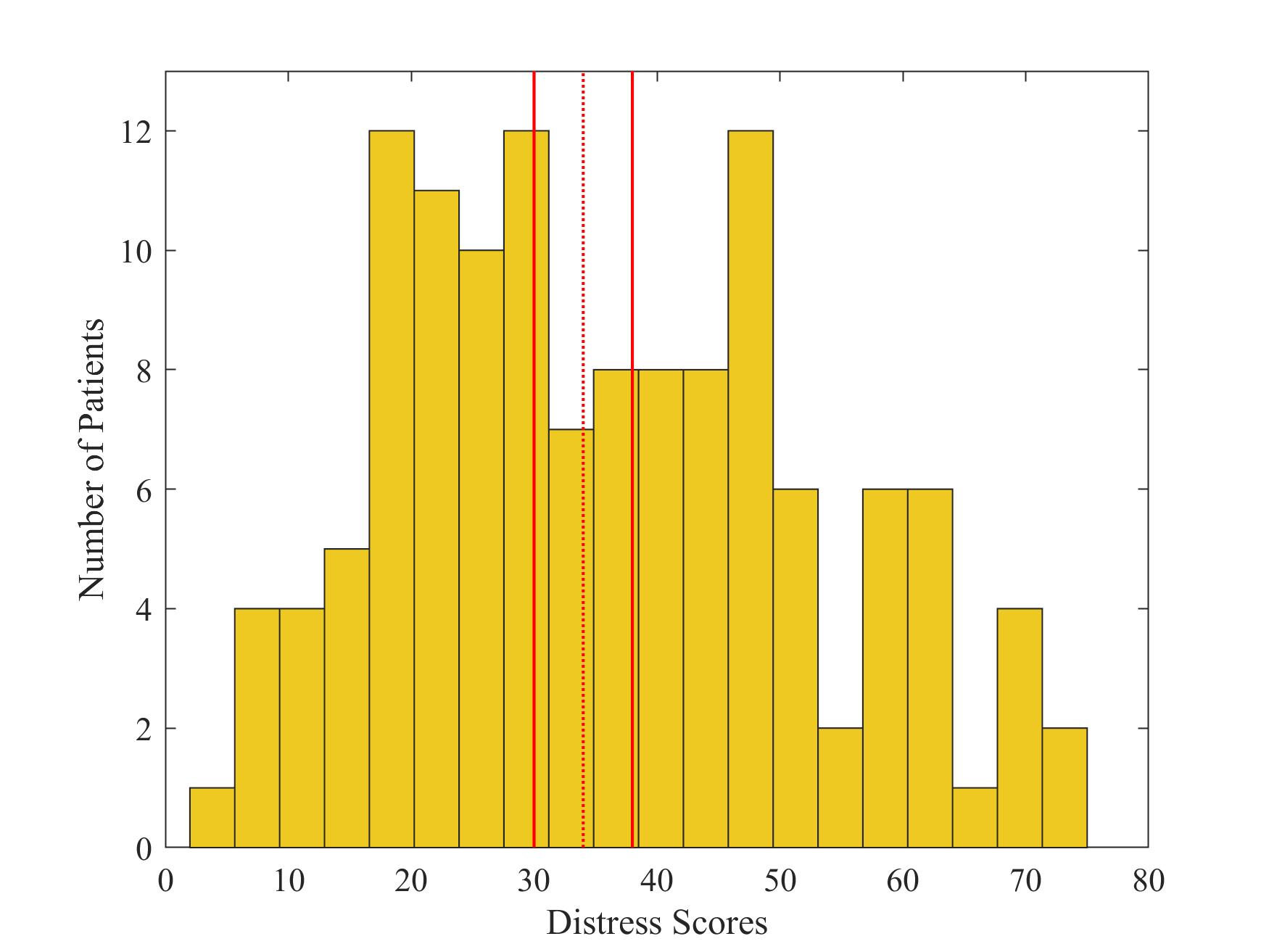


**Supplementary Figure 1.** Distribution of distress scores for the tinnitus patients. The solid red lines represent the thresholds for low and high distress characterizations, whereas the dotted red line shows the median of the distribution.

Tinnitus patients were divided in two non-overlapping groups based on their distress scores for the distress classifier. We excluded 22 patients with distress scores in the 30-38 interval. The interval of scores was chosen such that it was symmetric with respect to the distribution’s median value (34), hence retaining a total of 107 patients:

- **The low distress group** included patients with a distress score less than 30 (51 patients).
- **The high distress group** included patients with a distress score greater than 38 (56 patients).

| **Features** |  | **High Distress**  **(HD)** |  | **Low Distress**  **(LD)** |  | **High versus Low Distress Comparison** | | |
| --- | --- | --- | --- | --- | --- | --- | --- | --- |
|  |  |  |  |  |  | **statistics** |  | **significance** |
| **Age** (years) |  | ***52.3 ± 1.8*** |  | ***45.4 ± 2.2*** |  | ***t-val = 2.45*** |  | ***HD > LD, p < 0.02*** |
| **Gender**  (males - females) |  | 36 - 20 |  | 39 - 12 |  | Chi^2^* = 1.35 |  | NS, p < 0.25 |
| **Hearing Loss** (dB) |  | ***31.0 ± 2.5*** |  | ***16.6 ± 1.8*** |  | ***t-val = 4.56*** |  | ***HD > LD, p < 0.001*** |
| **Tinnitus Type**  (narrow band - pure tone) |  | 37 - 19 |  | 31 - 20 |  | Chi^2*^ = 0.13 |  | NS, p < 0.72 |
| **Tinnitus Side**  (unilateral - bilateral) |  | 44 -12 |  | 34 -17 |  | Chi^2*^ = 1.36 |  | NS, p < 0.25 |
| **Tinnitus Duration** (years) |  | *6.4 ± 1.0* |  | *4.3 ± 0.6* |  | *t-val = 1.78* |  | *HD > LD, p < 0.08* |
| **Tinnitus Frequency** (Hz) |  | 4853 ± 408 |  | 5033 ± 430 |  | t-val = 0.30 |  | NS, p < 0.77 |
| **Hearing Loss at Tinnitus Frequency** (dB) |  | ***49.5 ± 3.6*** |  | ***35.4 ± 3.6*** |  | ***t-val = 2.75*** |  | ***HD > LD, p < 0.01*** |
| **Loudness at Tinnitus Frequency** (sensation, dB) |  | 7.5 ± 1.2 |  | 8.5 ± 1.2 |  | t-val = -0.59 |  | NS, p < 0.56 |
| **Tinnitus Questionnaire: Distress** |  | ***52.1 ± 1.3*** |  | ***19.4 ± 0.9*** |  | ***t-val = 20.17*** |  | ***HD > LD, p <0.001*** |
| **Subjective Loudness (VAS)** |  | ***6.3 ± 0.3*** |  | ***4.1 ± 0.3*** |  | ***t-val = 5.66*** |  | ***HD > LD, p < 0.001*** |

**Supplementary Table 2.** Descriptive statistics of the high and low distress groups are presented with between-group comparisons for demographic and tinnitus features. Descriptive statistics are presented as mean ± standard error, except for categorical features where the exact numbers of patients in each category is reported. Between-group comparisons are conducted using the Chi^2*^ test with Yate’s correction (denoted as Chi^2*^ in the statistics column), for categorical features, while for continuous features, significance of comparisons is assessed using the unpaired t-test (denoted as t-val in the statistics column). Features showing significant between-group comparisons (p < 0.05) are written in bold italics, whereas those with a tendency towards significance are written in plain italics.

The high distress patients had significantly higher (p < 0.05) age, hearing loss level, hearing loss at tinnitus frequency, and subjective loudness (VAS) as compared to the low distress group. In accordance with guidelines from the World Health Organization, hearing loss for high distress patients was characterized by a slight impairment (26-40 dB), while the low distress group had no impairment (0-25 dB). For more information, see the document entitled “Health risks from exposure to noise from personal music players” from the Scientific Committee on Emerging and Newly Identified Health Risks (2008).


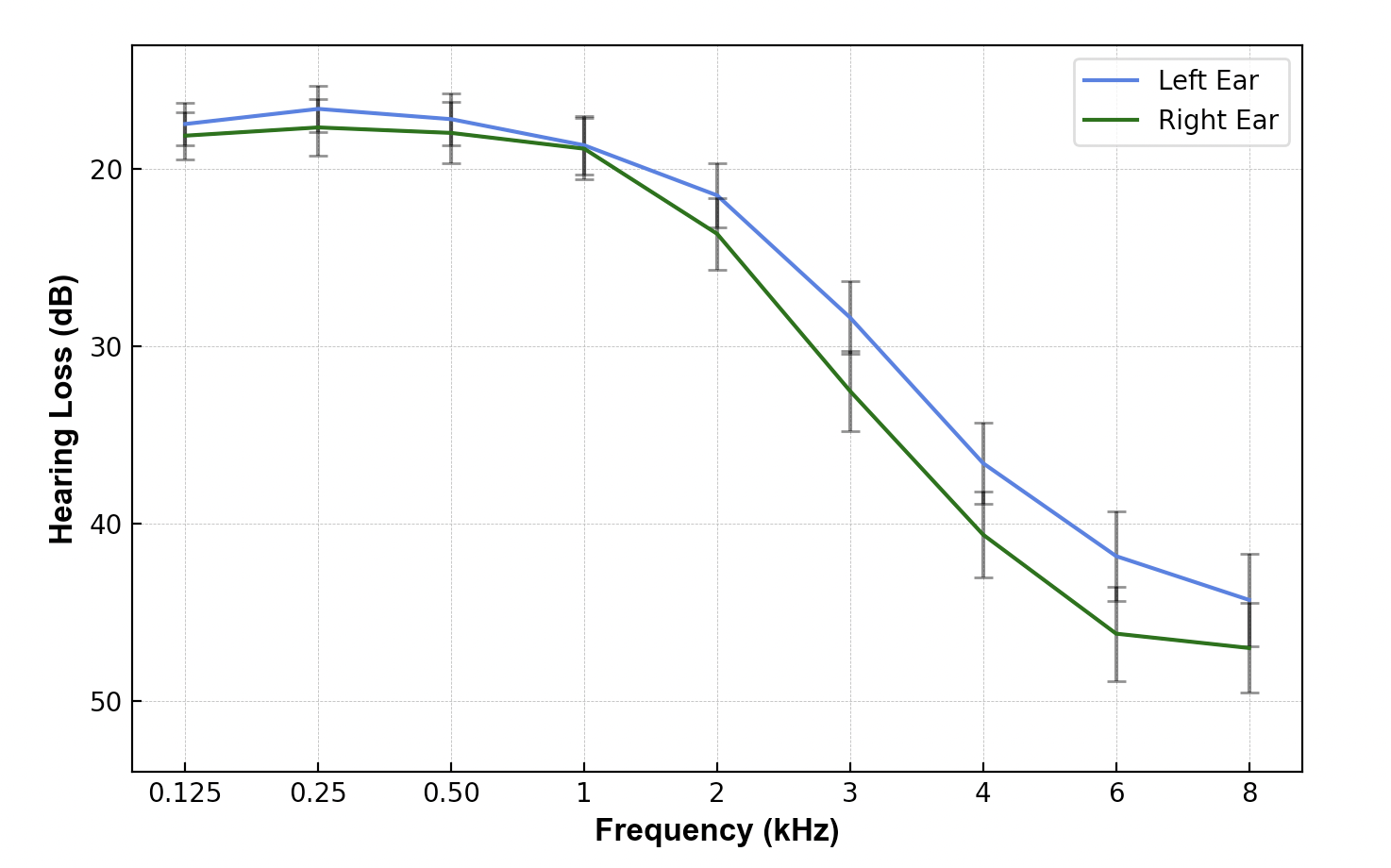


**Supplementary Figure 2. Audiogram results for tinnitus patients.** The level of hearing loss (dB) is plotted against tone frequency (kHz) for both the left and right ears. An audiogram test detects the threshold of hearing for a particular frequency. These results were obtained from all 129 tinnitus patients.

**Supplementary Appendix B: Tinnitus Patients vs. Control Classification**

**Desikan-Killiany Atlas Regions and Acronyms**

| **Region #** | **Cortical Area** | **Acronym** |
| --- | --- | --- |
| 1 | bankssts L | BSTS-l |
| 2 | bankssts R | BSTS-r |
| 3 | caudalanteriorcingulate L | CACC-l |
| 4 | caudalanteriorcingulate R | CACC-r |
| 5 | caudalmiddlefrontal L | CMF-l |
| 6 | caudalmiddlefrontal R | CMF-r |
| 7 | cuneus L | Cu-l |
| 8 | cuneus R | Cu-r |
| 9 | entorhinal L | Ent-l |
| 10 | entorhinal R | Ent-r |
| 11 | frontalpole L | FP-l |
| 12 | frontalpole R | FP-r |
| 13 | fusiform L | Fu-l |
| 14 | fusiform R | Fu-r |
| 15 | inferiorparietal L | IP-l |
| 16 | inferiorparietal R | IP-r |
| 17 | inferiortemporal L | IT-l |
| 18 | inferiortemporal R | IT-r |
| 19 | insula L | INS-l |
| 20 | insula R | INS-r |
| 21 | isthmuscingulate L | ISC-l |
| 22 | isthmuscingulate R | ISC-r |
| 23 | lateraloccipital L | LOC-l |
| 24 | lateraloccipital R | LOC-r |
| 25 | lateralorbitofrontal L | LOF-l |
| 26 | lateralorbitofrontal R | LOF-r |
| 27 | lingual L | Lin-l |
| 28 | lingual R | Lin-r |
| 29 | medialorbitofrontal L | MOF-l |
| 30 | medialorbitofrontal R | MOF-r |
| 31 | middletemporal L | MT-l |
| 32 | middletemporal R | MT-r |
| 33 | paracentral L | PCL-l |
| 34 | paracentral R | PCL-r |
| 35 | parahippocampal L | PHG-l |
| 36 | parahippocampal R | PHG-r |
| 37 | parsopercularis L | POP-l |
| 38 | parsopercularis R | POP-r |
| 39 | parsorbitalis L | POR-l |
| 40 | parsorbitalis R | POR-r |
| 41 | parstriangularis L | PTR-l |
| 42 | parstriangularis R | PTR-r |
| 43 | pericalcarine L | PCal-l |
| 44 | pericalcarine R | PCal-r |
| 45 | postcentral L | PSC-l |
| 46 | postcentral R | PSC-r |
| 47 | posteriorcingulate L | PCC-l |
| 48 | posteriorcingulate R | PCC-r |
| 49 | precentral L | PRC-l |
| 50 | precentral R | PRC-r |
| 51 | precuneus L | PCu-l |
| 52 | precuneus R | PCu-r |
| 53 | rostralanteriorcingulate L | RACC-l |
| 54 | rostralanteriorcingulate R | RACC-r |
| 55 | rostralmiddlefrontal L | RMF-l |
| 56 | rostralmiddlefrontal R | RMF-r |
| 57 | superiorfrontal L | SF-l |
| 58 | superiorfrontal R | SF-r |
| 59 | superiorparietal L | SP-l |
| 60 | superiorparietal R | SP-r |
| 61 | superiortemporal L | ST-l |
| 62 | superiortemporal R | ST-r |
| 63 | supramarginal L | SMG-l |
| 64 | supramarginal R | SMG-r |
| 65 | temporalpole L | TP-l |
| 66 | temporalpole R | TP-r |
| 67 | transversetemporal L | TT-l |
| 68 | transversetemporal R | TT-r |

**Supplementary Table 3.** Cortical areas from the Desikan-Killiany atlas are presented along with their numerical identifiers and corresponding acronyms as a guide for readers to reference^1-5^.

**Classification Procedure**

**Feature Collection**

The following features were taken into account^6-8^:

1. Power Spectral Density (PSD)
2. Relative PSD
3. Lempel-Ziv Complexity (LZC)
4. Inward Flow of Information
5. Outward Flow of Information
6. Graph Theory Metrics

Features 1 to 5 were estimated for each band of interest (delta, theta, alpha, beta1, beta2, and gamma) and cortical areas (68 areas based on Desikan Killiany Atlas^5^, see Supplementary Table 3).

For each band of interest, five graph theory metrics were estimated to characterize the network’s behaviour^8^:

- Clustering Coefficient
- Global Efficiency
- Network Strength
- In-Degree Participation Coefficient
- Out-Degree Participation Coefficient

Altogether, we obtained 36 datasets: 30 datasets (features 1-5 for each of the 6 bands) included 68 values, and the other six included five values (feature 6, five graph metrics).

**Features Selection**

The feature selection procedure was divided into three-steps:

1. **Dataset selection procedure.** Each dataset was submitted to a between-group (tinnitus versus controls) comparison based on unpaired t-tests. For features 1 to 5, 68 between-group comparisons (one per cortical area) were conducted for each band, while for feature 6, 5 between-group comparisons were conducted for each band (one per graph theory metric). Uncorrected significance was used and set at p = 0.05 (corresponding to t = |1.97|). Only datasets that showed at least one significant difference were retained for further analyses.
2. **Features selection procedure.** The features of each retained dataset were standardized using a z-score transformation and then submitted to a neighbourhood component analysis. The analysis was run 1000 times: at each step, the weights associated with the 68 features (5 for graph theory metrics) were obtained. The weights of a feature reflect its contribution to discriminating between the groups. At the end of the procedure, the mean weight of each feature was estimated as the average over the 1000 runs. The algorithm was run multiple times to ensure quantitative stability for the weights obtained. For each dataset, only those features with higher between-group discriminatory power were selected based on an empirically chosen threshold. The threshold value was chosen to balance high discriminatory ability with the number of features to be used for the SVM classifier. The selected features were then merged into a single group. The effectiveness of the selected features was then verified by using them to train and validate a linear SVM with 5-folds cross-validation. The procedure was run 1000 times, and the accuracy was obtained for each run. Finally, the classifier’s accuracy was estimated by averaging over the 1000 runs.
3. **Feature pruning and optimization.** Once the effectiveness of selected features in correctly classifying the subjects of the two groups was verified, we reduced the number of features with the aim of optimizing the datasets while also enhancing the classifier’s performances: a step-down procedure was used to eliminate redundant parameters while controlling for the SVM performance by monitoring the validation accuracy levels. At each pruning step, the linear SVM with 5-folds cross-validation was run 1000 times and its accuracy was estimated by averaging over the 1000 runs.

**Dataset Selection for PSD**


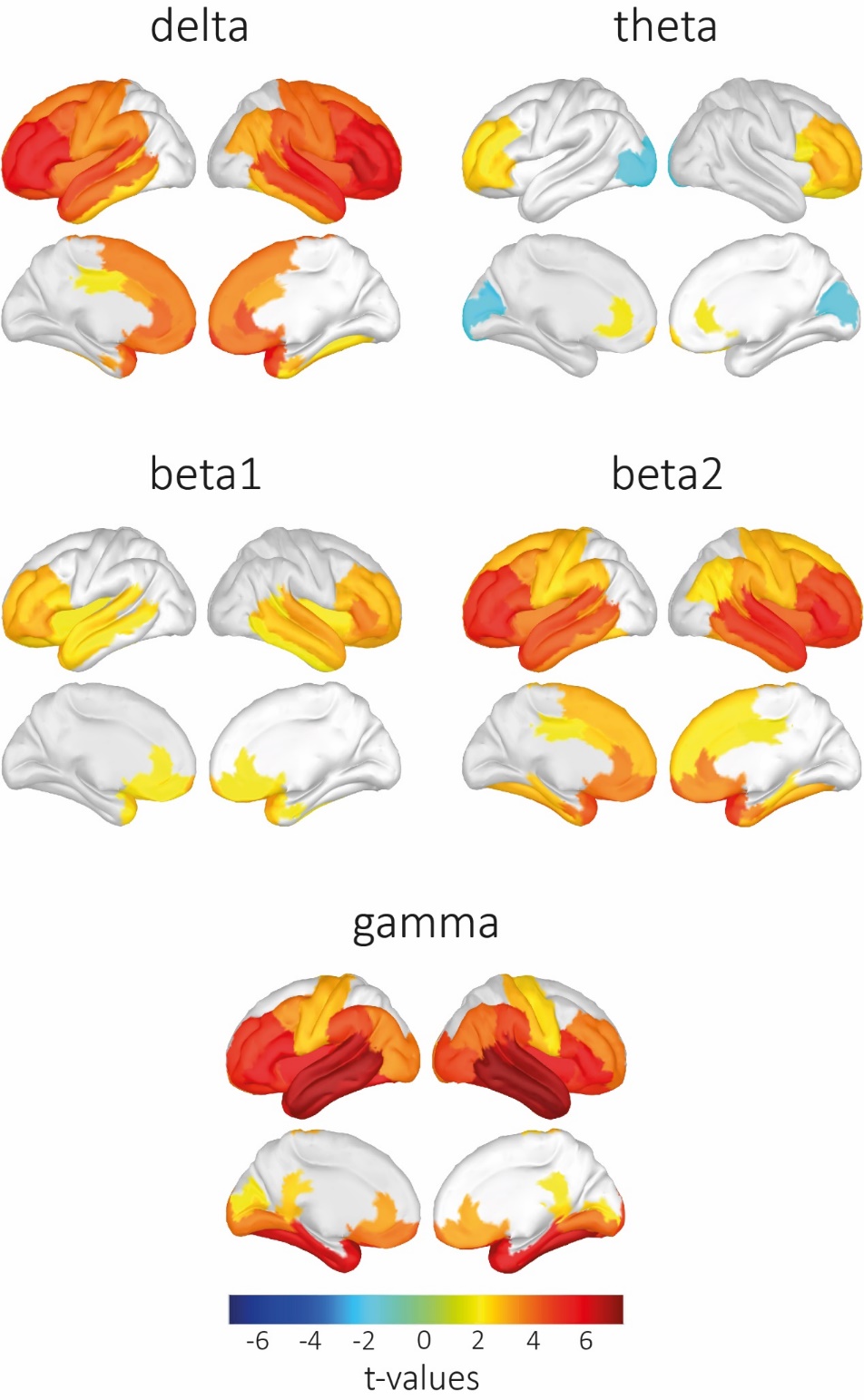


**Supplementary Figure 3. Band-wise PSD comparison:** Cortical maps with regional t-values are given for the bands that had at least one area showing a significant difference between tinnitus patients and controls. Regions not showing significant differences are left uncoloured. Between-group comparisons (tinnitus patients vs controls) for each band and area are conducted using unpaired t-tests.

**Feature Selection for PSD**


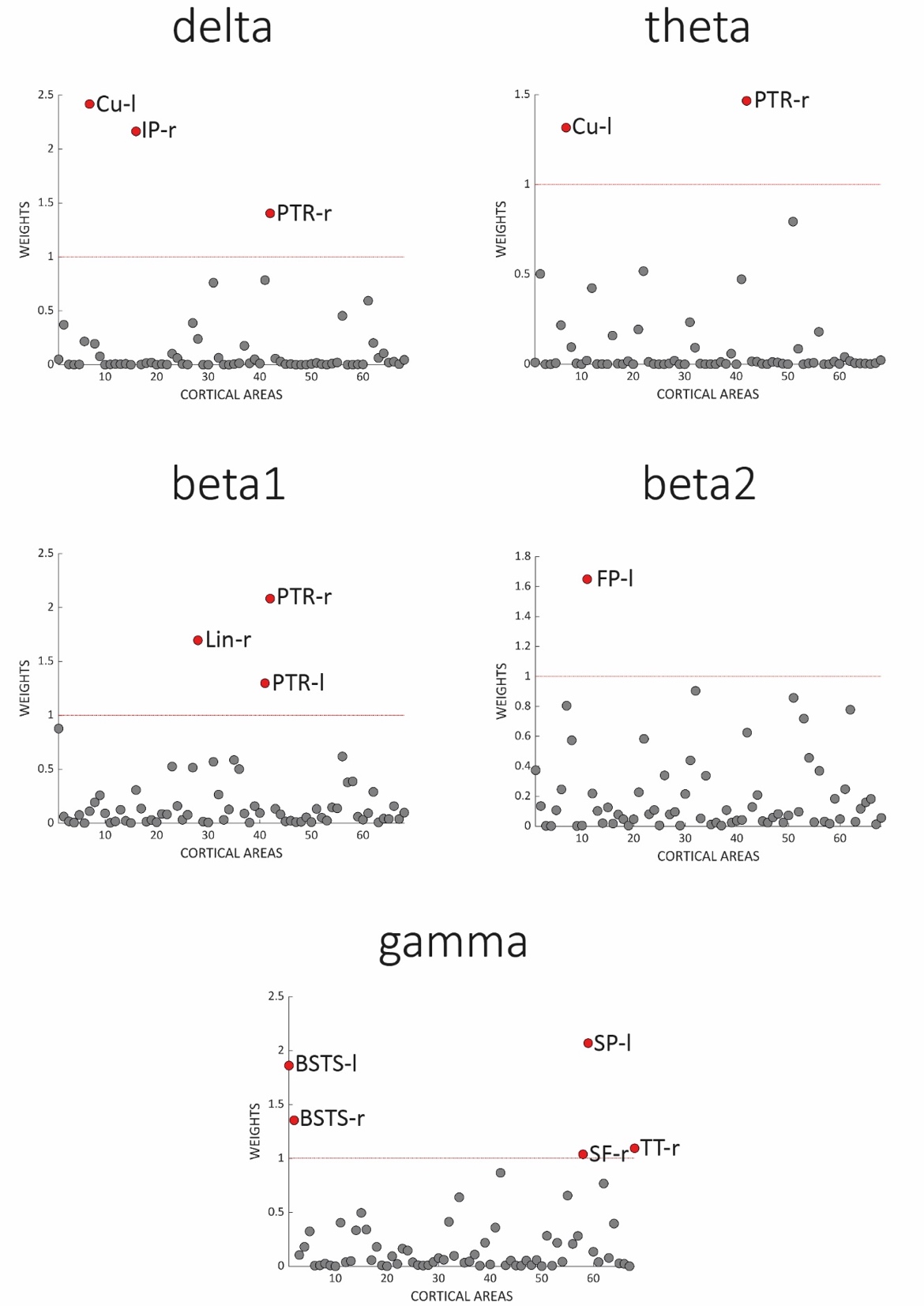


**Supplementary Figure 4. Bandwise PSD features:** For each band retained in the relative PSD feature, a scatterplot is presented to depict weights for the dataset’s cortical areas. The dashed red line represents the feature’s selection threshold, while red dots indicate the areas in which the feature satisfies the threshold. The red dots are also accompanied by the acronyms of their corresponding areas.

**Dataset Selection for Relative PSD**


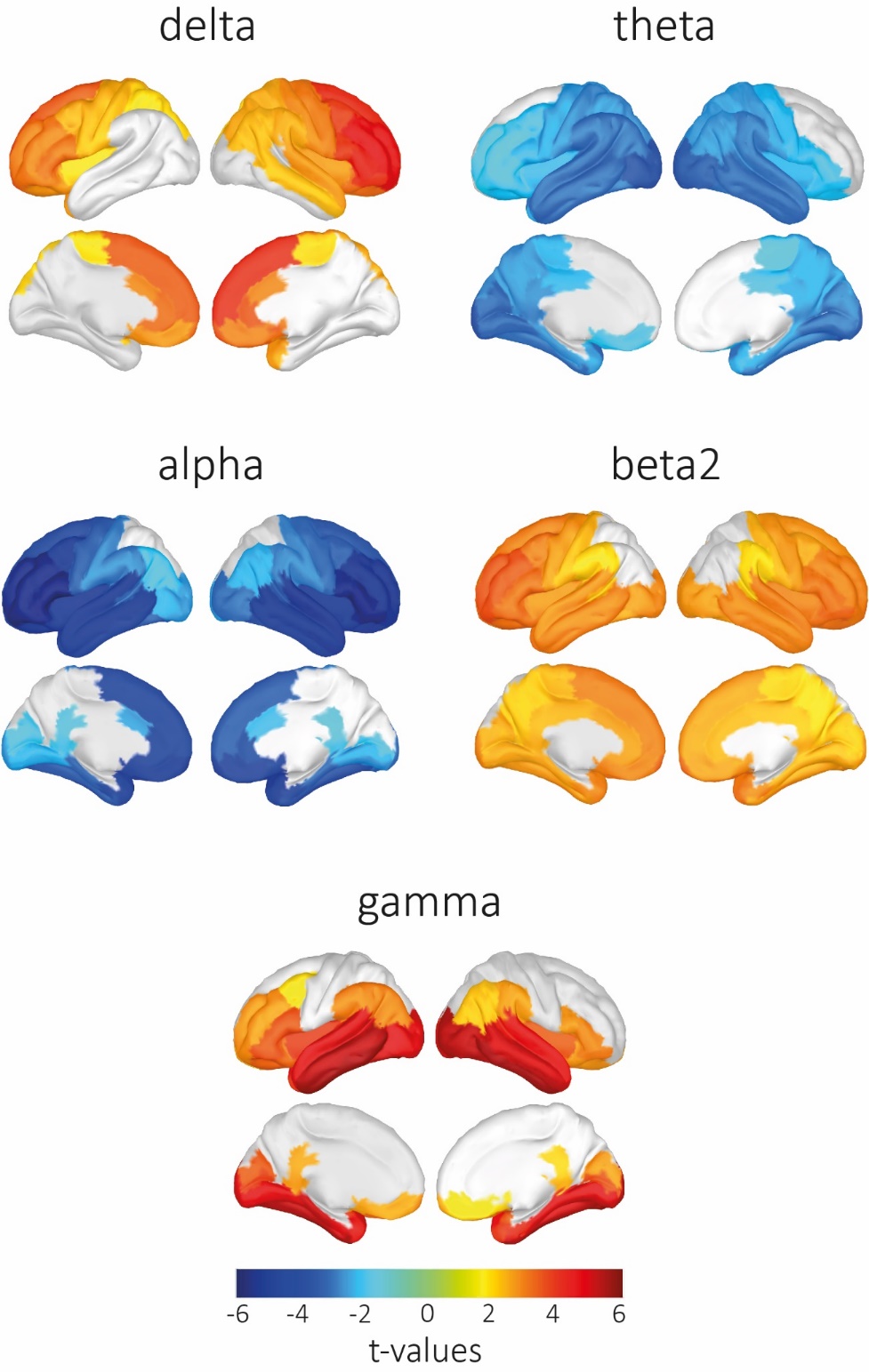


**Supplementary Figure 5. Band-wise relative PSD comparison:** Cortical maps with regional t-values are given for the bands that had at least one area showing a significant difference between tinnitus patients and controls. Regions not showing significant differences are left uncoloured. Between-group comparisons (tinnitus patients vs controls) for each band and area are conducted using unpaired t-tests.

**Feature Selection for Relative PSD**


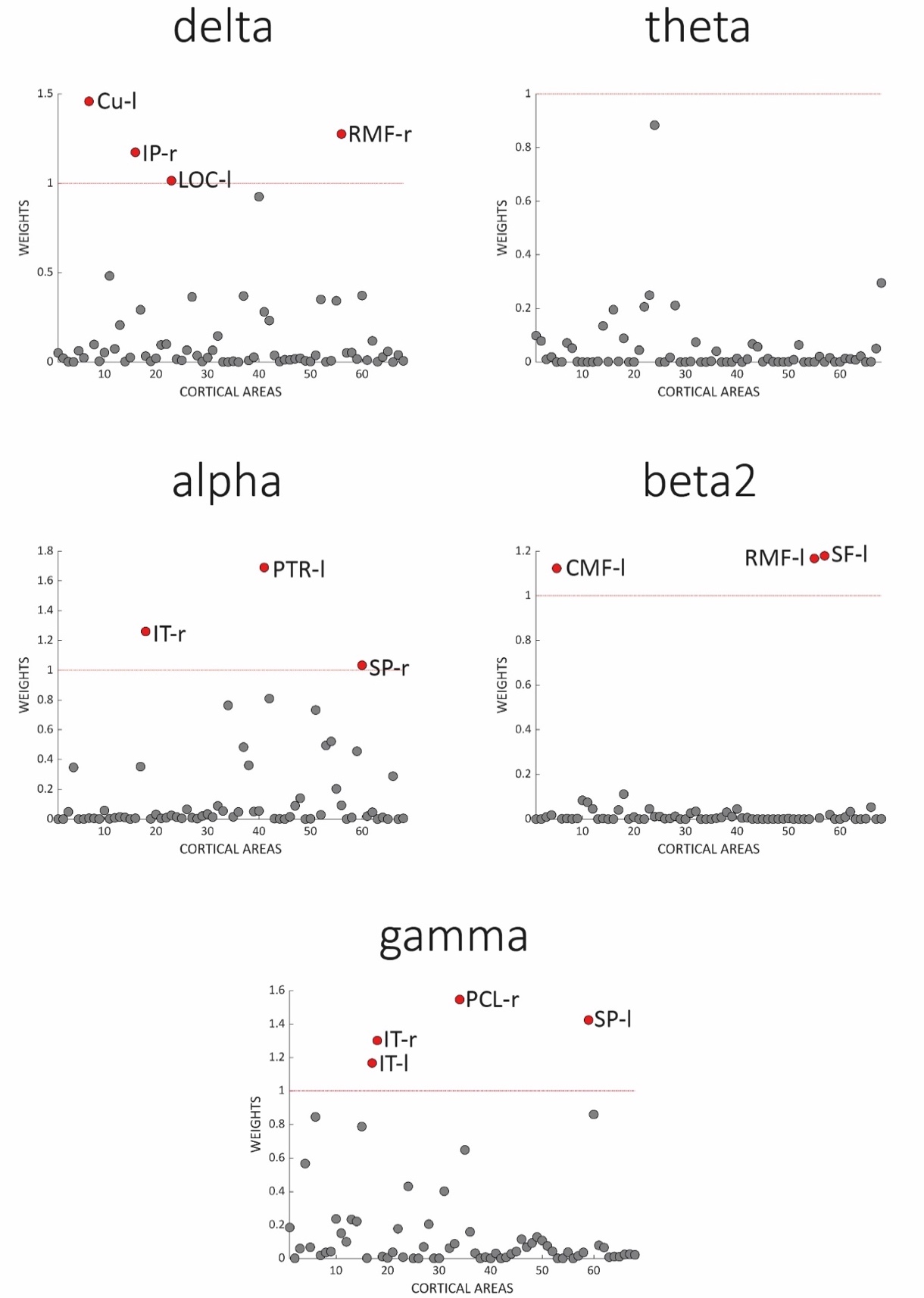


**Supplementary Figure 6. Band-wise relative PSD features:** For each band retained in the relative PSD feature, a scatterplot is presented to depict weights for the dataset’s cortical areas. The dashed red line represents the feature’s selection threshold, while red dots indicate the areas in which the feature satisfies the threshold. The red dots are also accompanied by the acronyms of their corresponding areas.

**Dataset Selection for LZC**


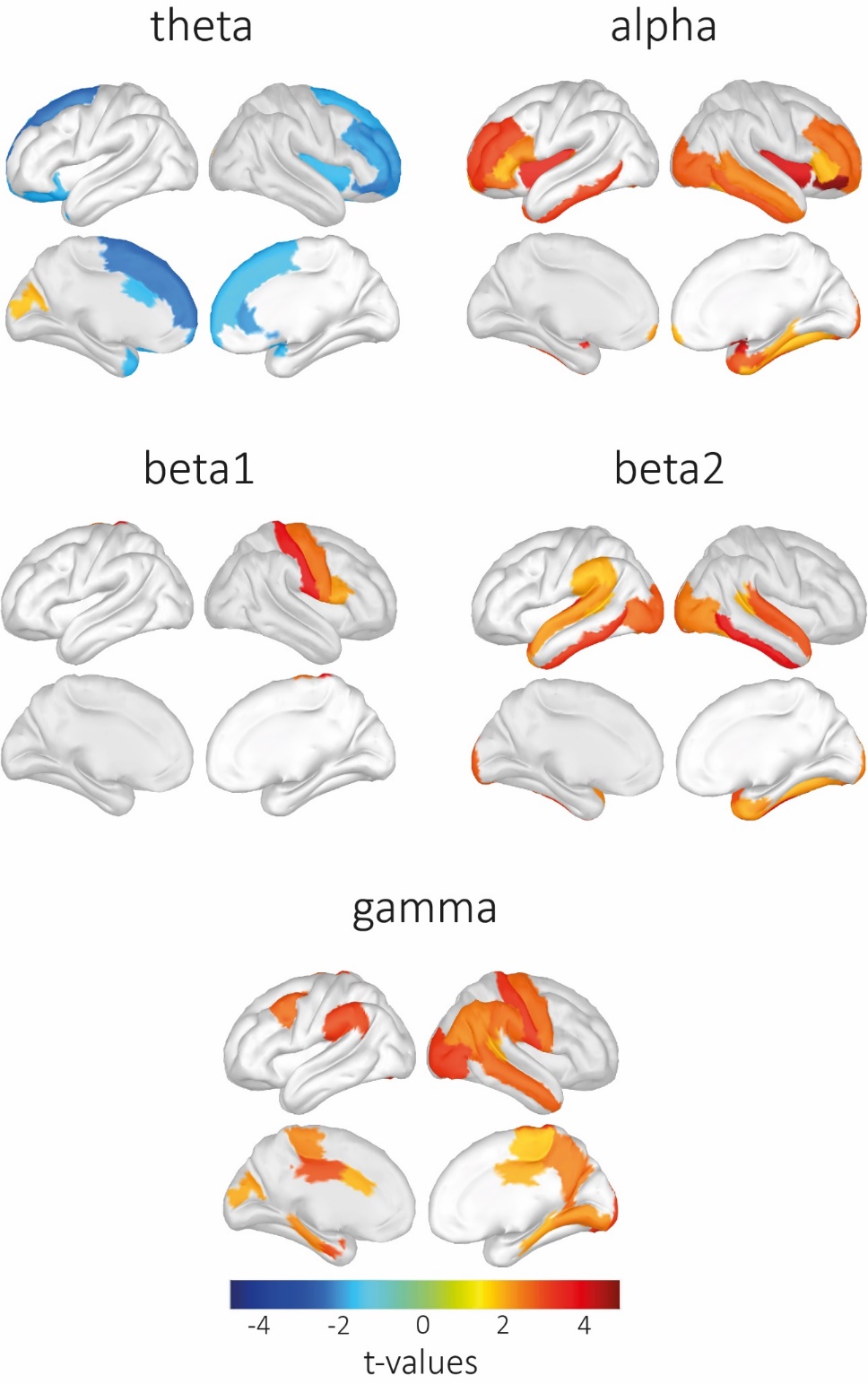


**Supplementary Figure 7. Band-wise LZC comparison:** Cortical maps with regional t-values are given for the bands that had at least one area showing a significant difference between tinnitus patients and controls. Regions not showing significant differences are left uncoloured. Between-group comparisons (tinnitus patients vs controls) for each band and area are conducted using unpaired t-tests.

**Feature Selection for LZC**


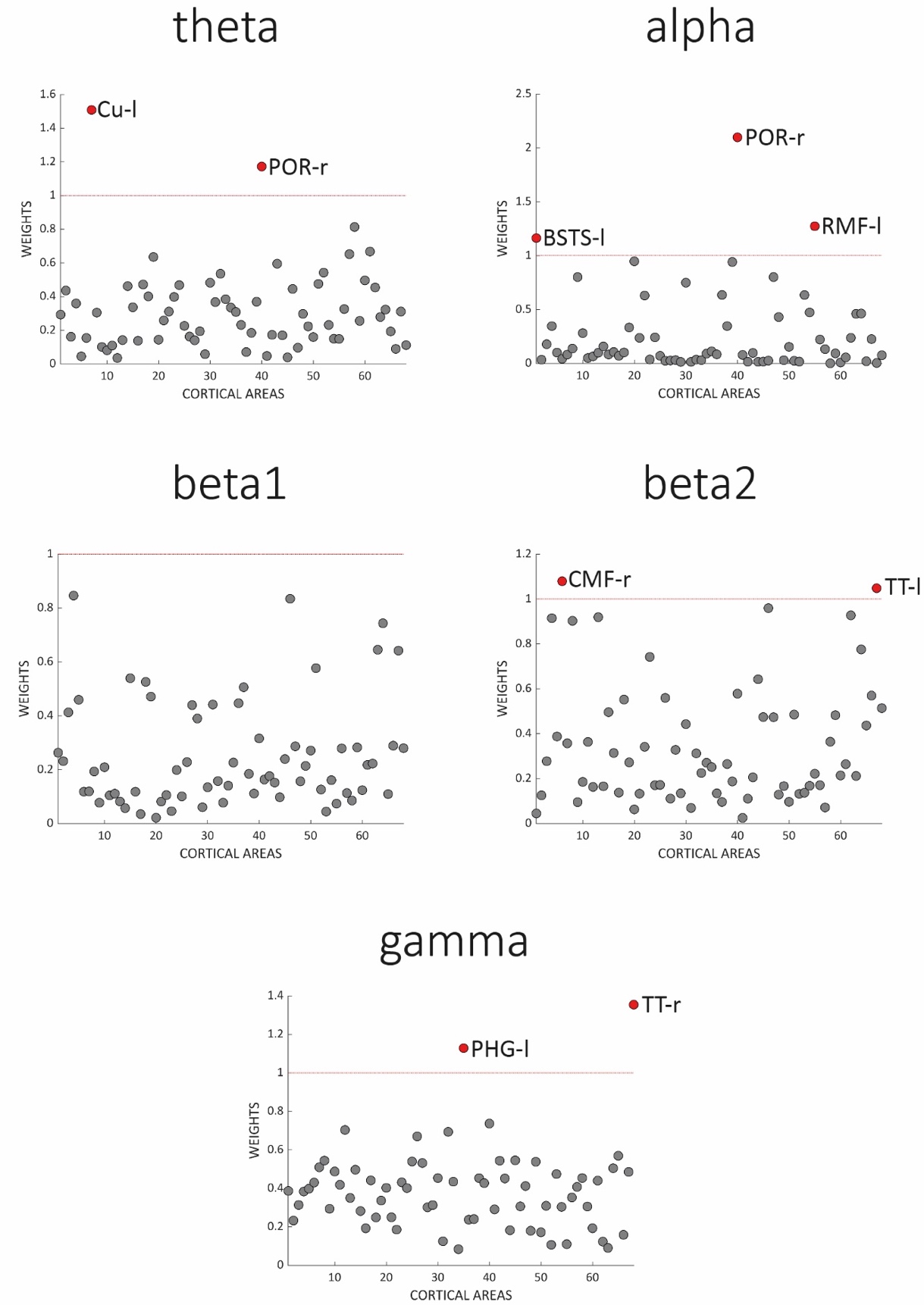


**Supplementary Figure 8. Band-wise LZC features:** For each band retained in the LZC feature, a scatterplot is presented to depict weights for the dataset’s cortical areas. The dashed red line represents the feature’s selection threshold, while red dots indicate the areas in which the feature satisfies the threshold. The red dots are also accompanied by the acronyms of their corresponding areas.

**Dataset Selection for Inward Flow of Information**


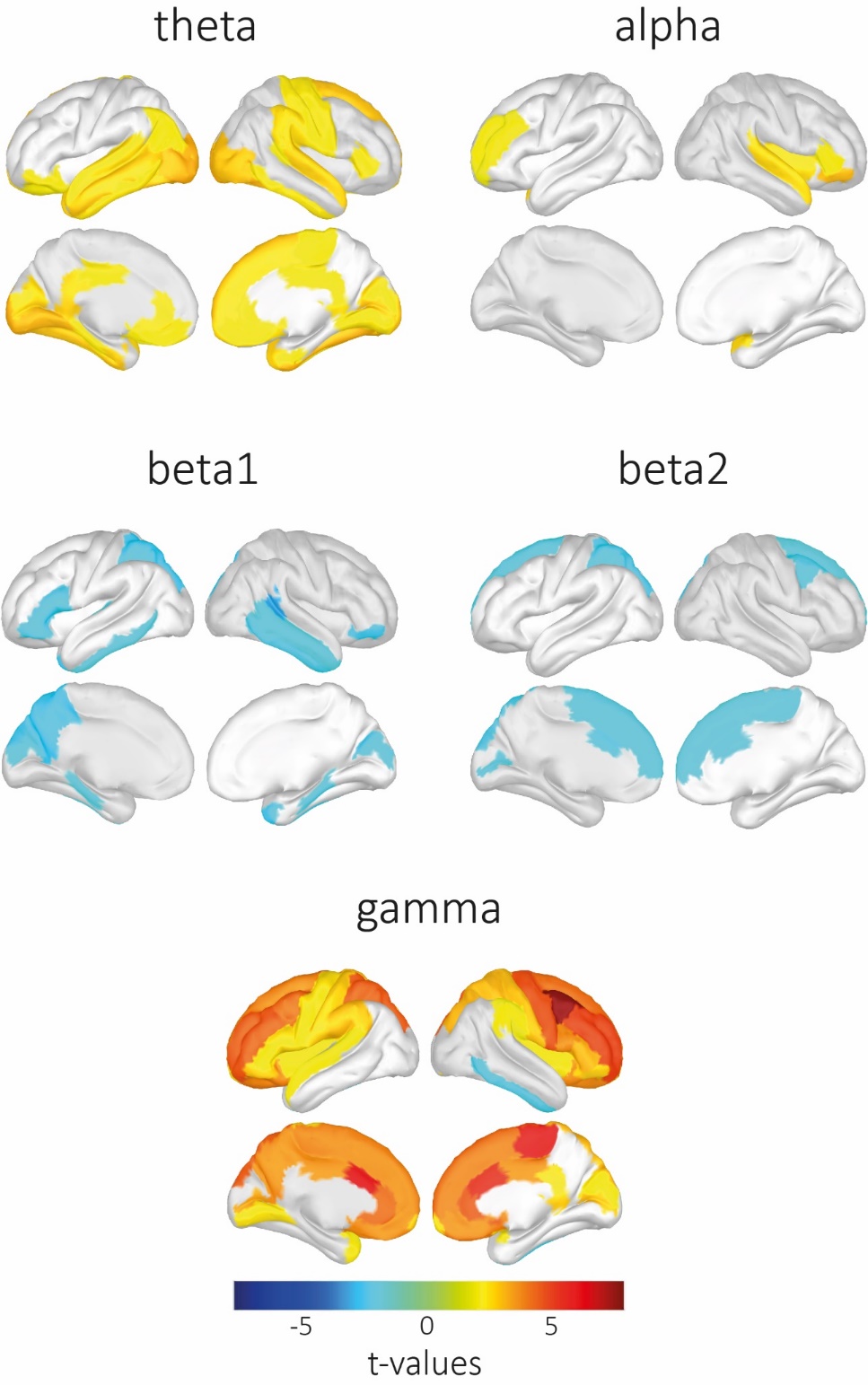


**Supplementary Figure 9. Band-wise inward flow of information comparison:** Cortical maps with regional t-values are given for the bands that had at least one area showing a significant difference between tinnitus patients and controls. Regions not showing significant differences are left uncoloured. Between-group comparisons (tinnitus patients vs controls) for each band and area are conducted using unpaired t-tests.

**Feature Selection for Inward Flow of Information**


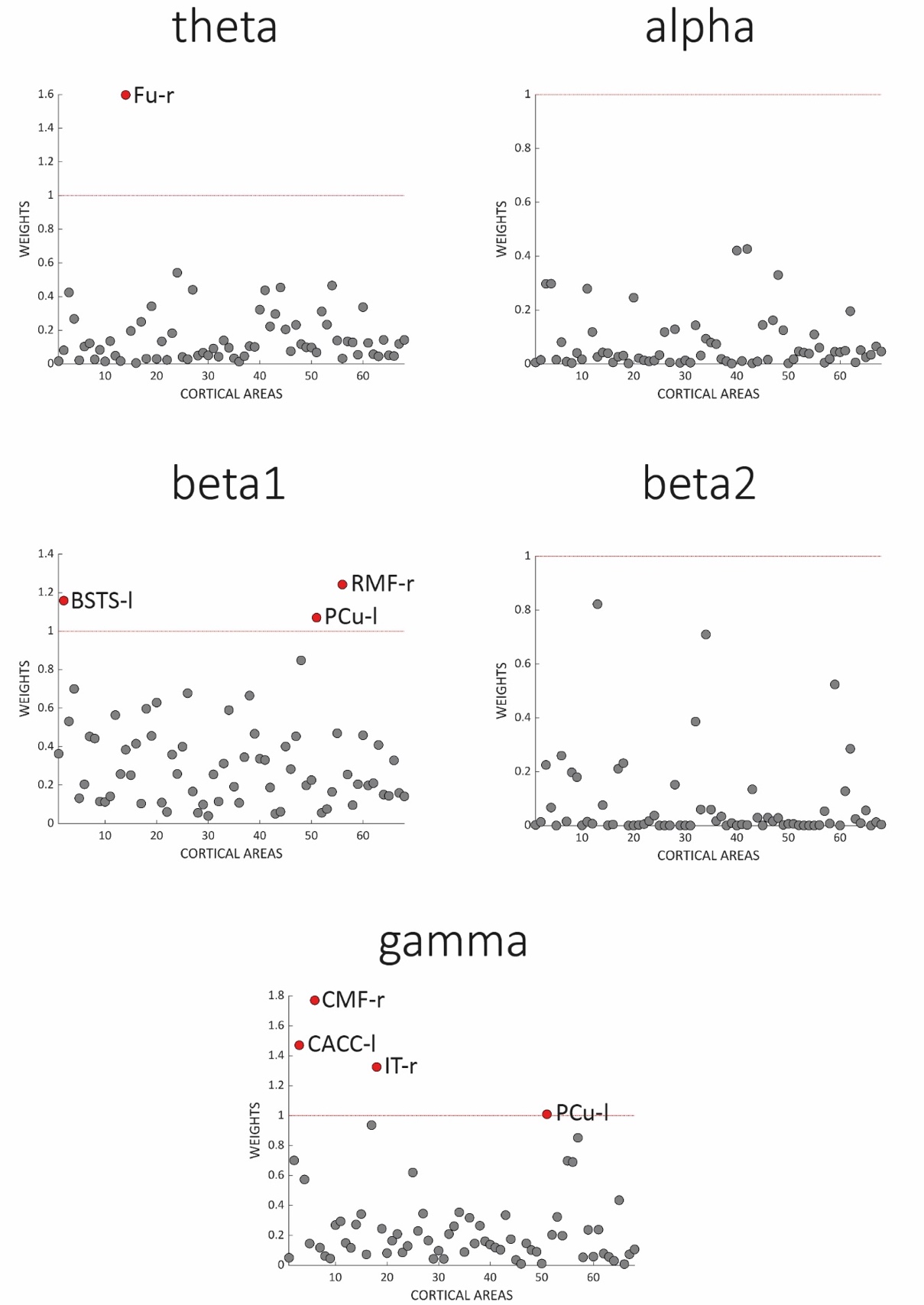


**Supplementary Figure 10. Band-wise inward flow of information features:** For each band retained in the inward flow of information feature, a scatterplot is presented to depict weights for the dataset’s cortical areas. The dashed red line represents the feature’s selection threshold, while red dots indicate the areas in which the feature satisfies the threshold. The red dots are also accompanied by the acronyms of their corresponding areas.

**Dataset Selection for Outward Flow of Information**


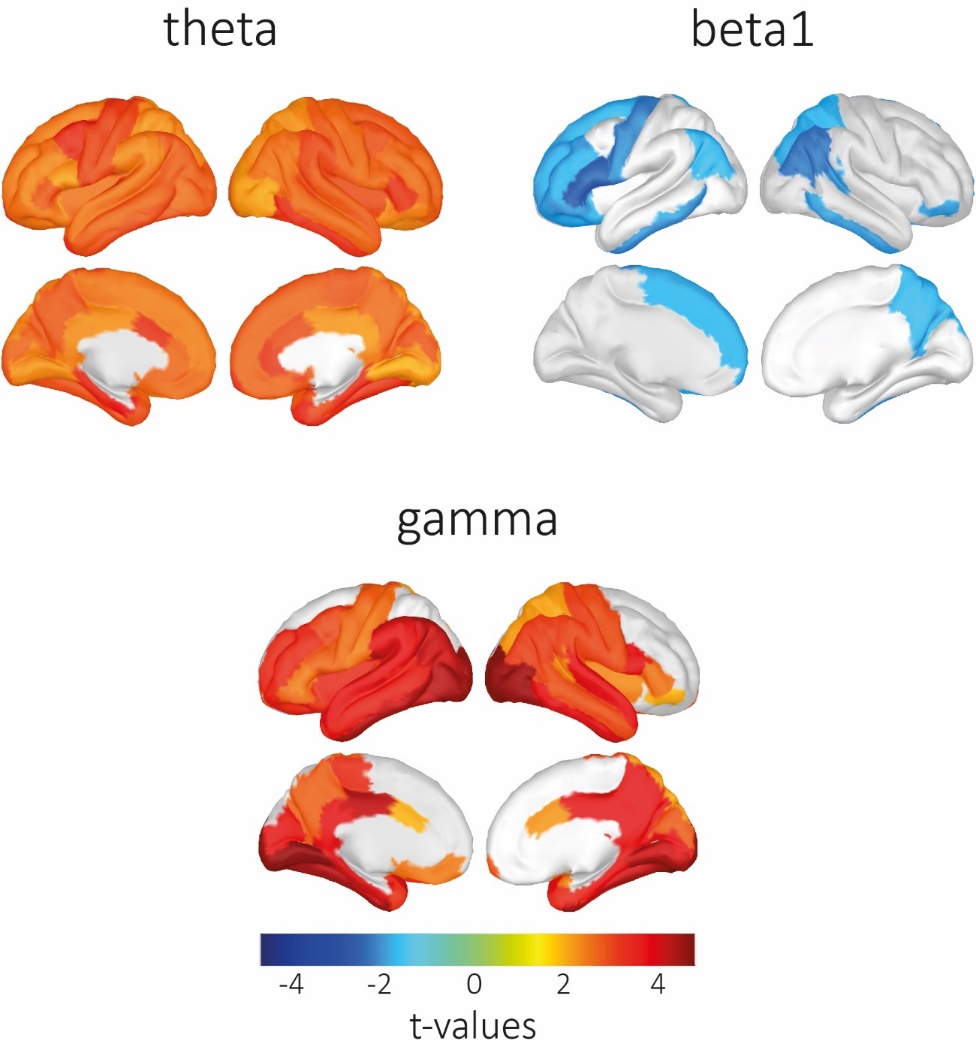


**Supplementary Figure 11. Band-wise outward flow of information comparison:** Cortical maps with regional t-values are given for the bands that had at least one area showing a significant difference between tinnitus patients and controls. Regions not showing significant differences are left uncoloured. Between-group comparisons (tinnitus patients vs controls) for each band and area are conducted using unpaired t-tests.

**Feature Selection for Outward Flow of Information**


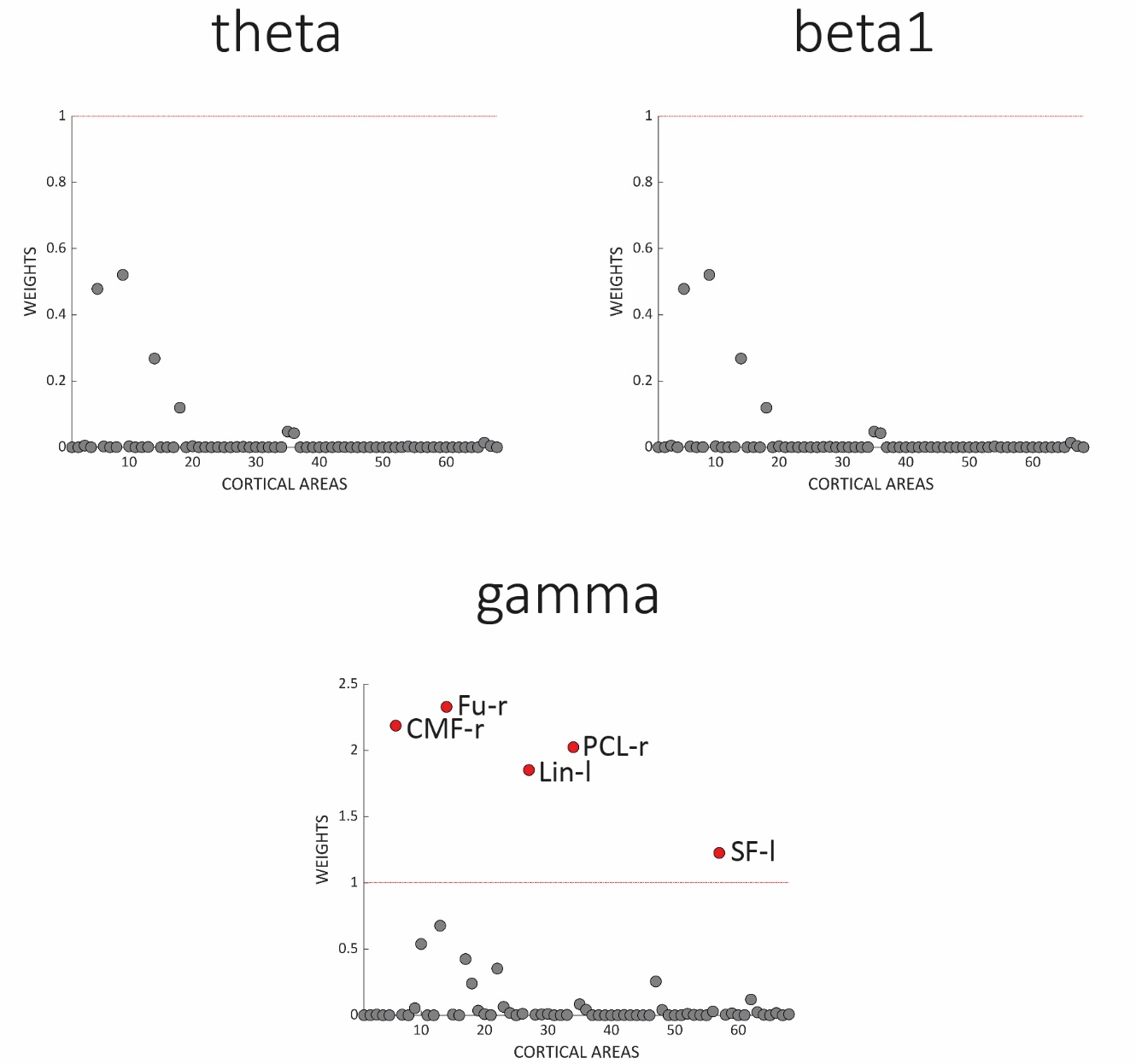


**Supplementary Figure 12. Band-wise outward flow of information features:** For each band retained in the outward flow of information feature, a scatterplot is presented to depict weights for the dataset’s cortical areas. The dashed red line represents the feature’s selection threshold, while red dots indicate the areas in which the feature satisfies the threshold. The red dots are also accompanied by the acronyms of their corresponding areas.

**Dataset Selection for Graph Theory Metrics**

|  | **CC** | | **GE** | | **NS** | | **MOD** | | **ID-PC** | | **OD-PC** | |
| --- | --- | --- | --- | --- | --- | --- | --- | --- | --- | --- | --- | --- |
|  | t-val | p-val | t-val | p-val | t-val | p-val | t-val | p-val | t-val | p-val | t-val | p-val |
| **delta** | -0.60 | 0.55 | ***-2.13*** | ***0.04*** | -1.7 | 0.24 | ***-4.09*** | ***0.001*** | ***-4.17*** | ***0.001*** | -0.90 | 0.37 |
| **theta** | ***3.77*** | ***0.001*** | ***2.53*** | ***0.02*** | ***2.83*** | ***0.005*** | 1.35 | 0.18 | 0.22 | 0.83 | 0.99 | 0.32 |
| **alpha** | 0.53 | 0.60 | 1.10 | 0.28 | 0.63 | 0.53 | 1.24 | 0.22 | 0.26 | 0.80 | 0.27 | 0.79 |
| **beta1** | 0.71 | 0.48 | ***2.26*** | ***0.03*** | 1.87 | 0.07 | -0.17 | 0.87 | 0.57 | 0.58 | 0.47 | 0.64 |
| **beta2** | 1.15 | 0.26 | ***2.80*** | ***0.006*** | 0.91 | 0.37 | ***-3.70*** | ***0.001*** | ***2.33*** | ***0.02*** | -0.50 | 0.62 |
| **gamma** | ***2.11*** | ***0.04*** | ***3.67*** | ***0.001*** | ***2.92*** | ***0.004*** | ***4.73*** | ***0.001*** | *2.27* | *0.03* | -0.07 | 0.95 |

**Supplementary Table 4.** For each band and graph theory metric, the t-value and corresponding p-value related to the tinnitus versus controls comparisons are reported (comparisons were conducted using unpaired t-tests). The statistics of features showing significant between-group differences are written in italics. (From here onwards, CC stands for Clustering Coefficient, GE for Global Efficiency, NS for Network Strength, MOD for Modularity, ID-PC for In-Degree Participation Coefficient and OD-PC for Out-Degree Participation Coefficient).

**Feature Selection for Graph Theory Metrics**


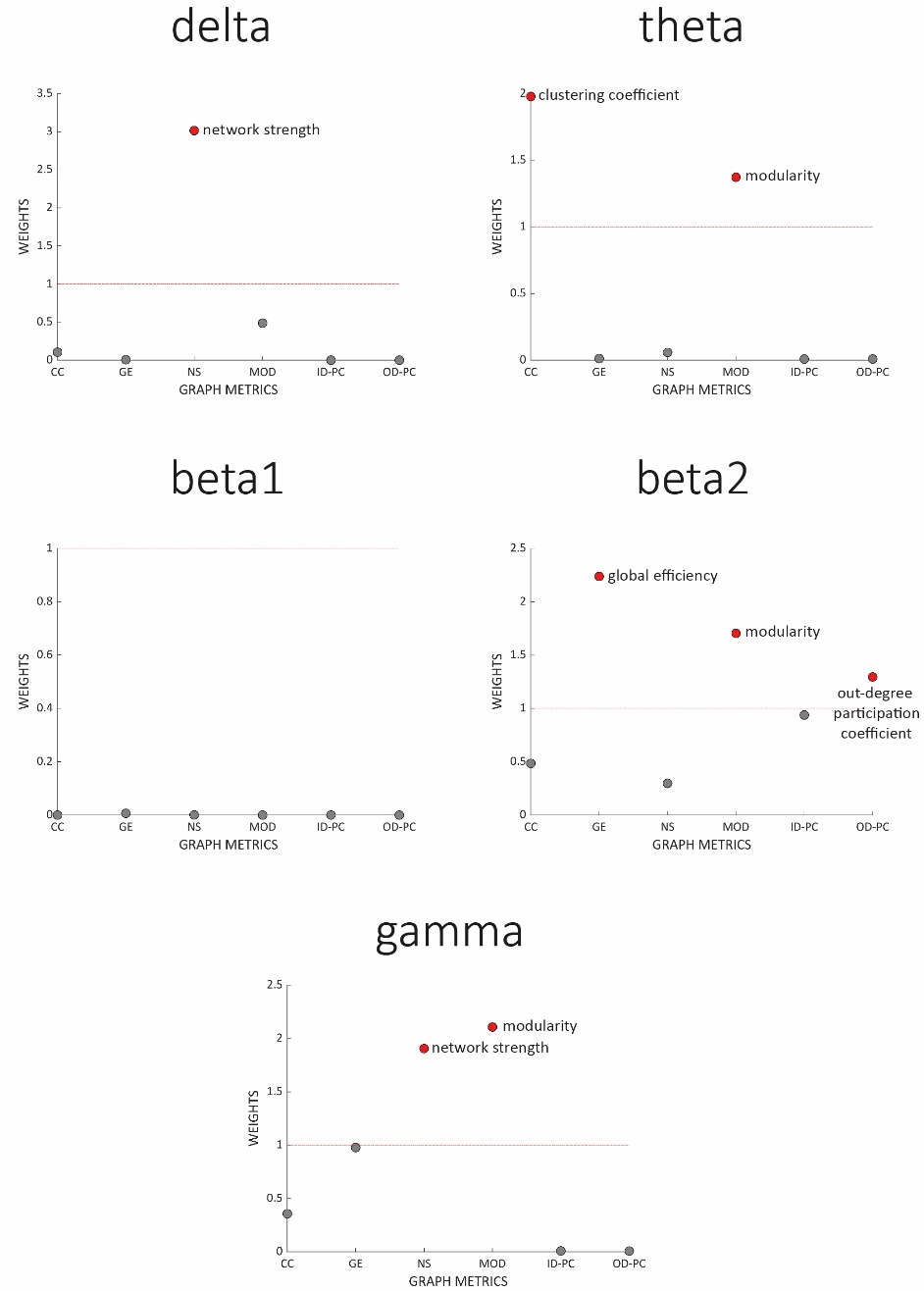


**Supplementary Figure 13. Graph theory features:** For each retained band, a scatterplot is presented to depict weights for all graph theory metrics. The dashed red line represents the feature’s selection threshold, while red dots indicate the metrics that are above the threshold.

**Pruning and Optimization of Feature Set**


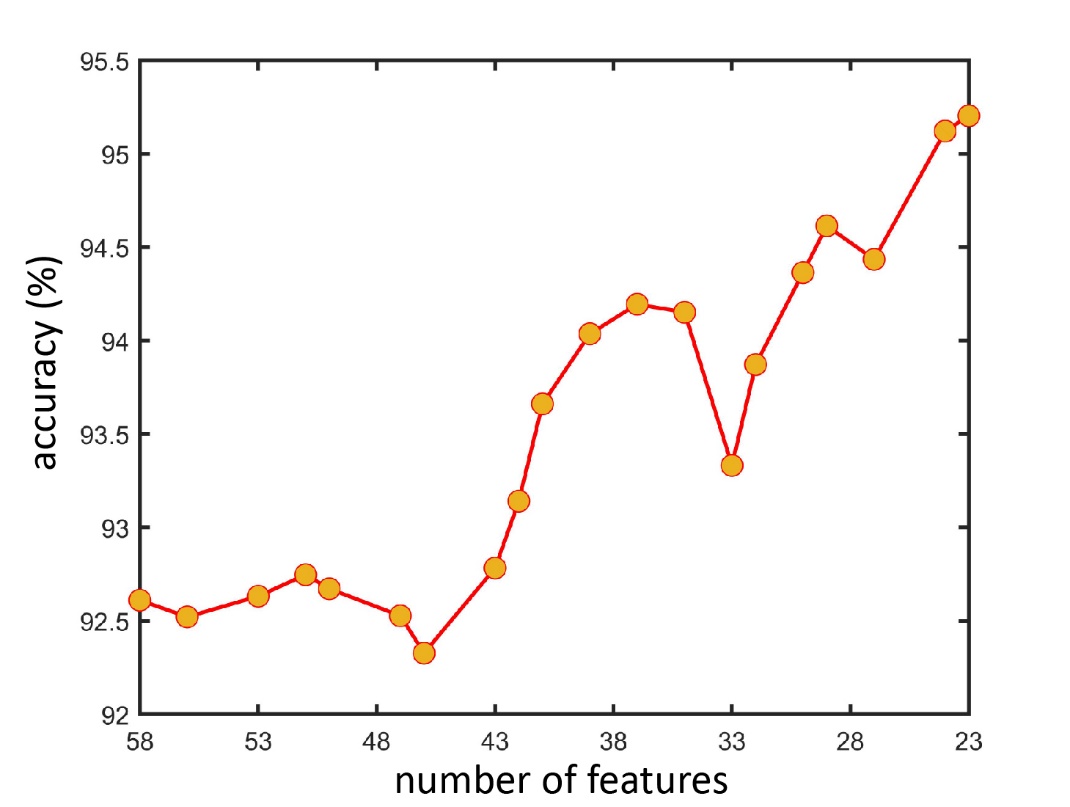


**Supplementary Figure 14.** The graph above shows the accuracy of the classifier versus the number of features used for it, which is decreasing from left to right in accordance with the pruning procedure.

**Final Features in Tinnitus vs. Control Classifier**

At the end of the features pruning and optimization procedure, which started with 58 features and a classification accuracy of 92.6%, we retained 23 features and reached an accuracy of 95.2%. In the figures below, we show the features retained for each dataset (PSD, relative PSD, LZC, Inward Flow of Information, and Outward Flow of Information) after the optimization procedure. On each cortical map, retained areas are identified in yellow. Each area is identified by its acronym and the frequency band(s) involved are specified in brackets.


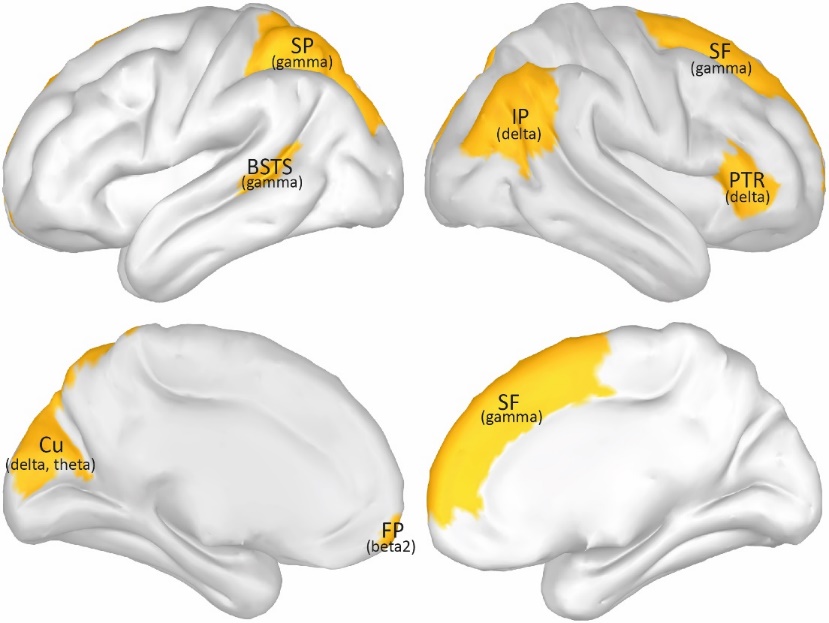


**Supplementary Figure 15. PSD**


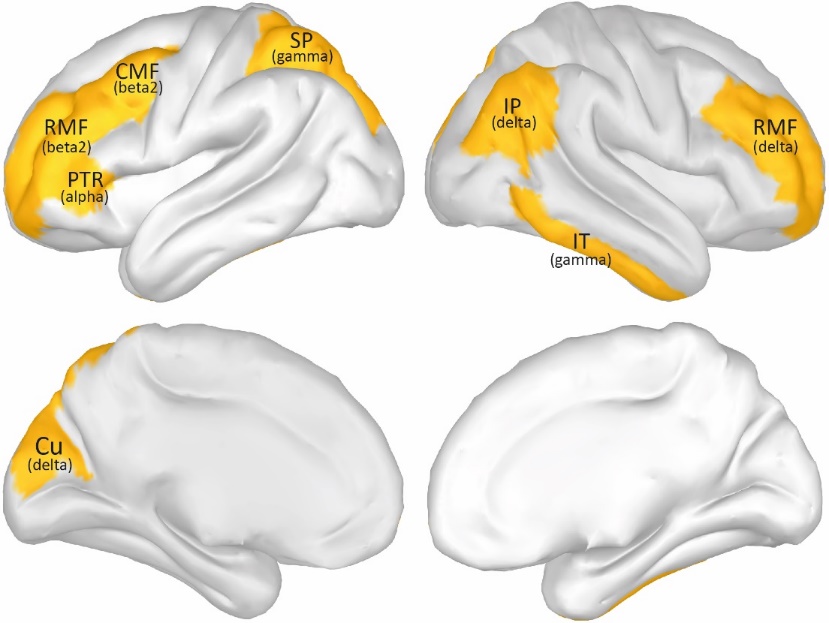


**Supplementary Figure 16. Relative PSD**


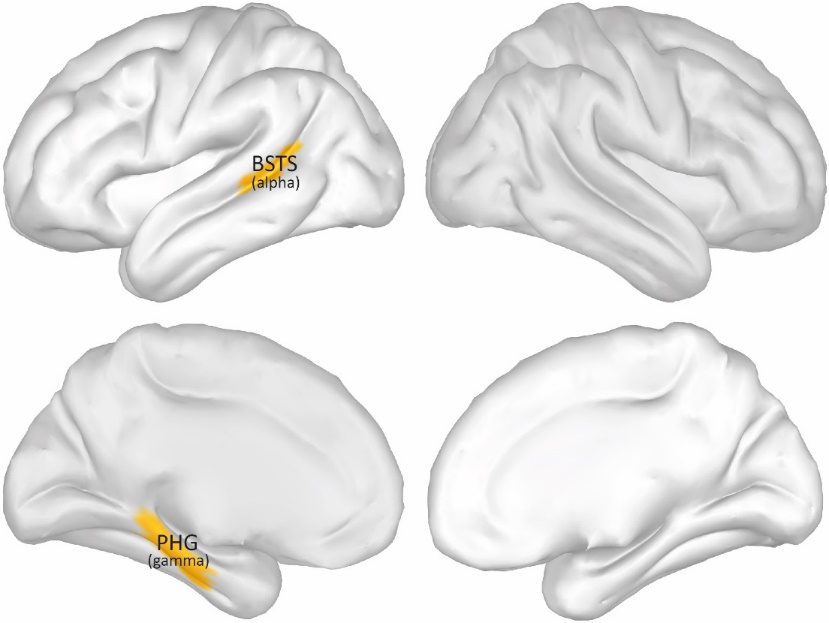


**Supplementary Figure 17. LZC**


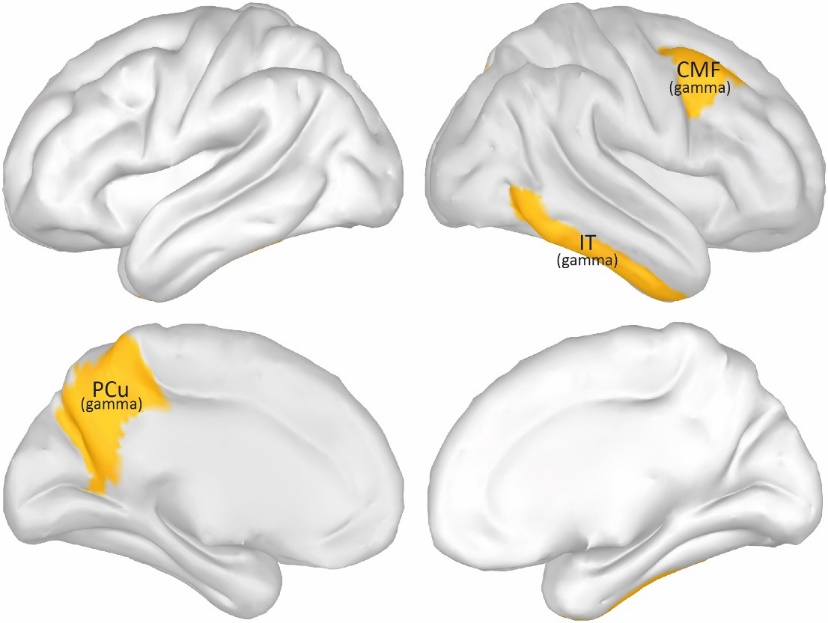


**Supplementary Figure 18. Inward Flow of Information**


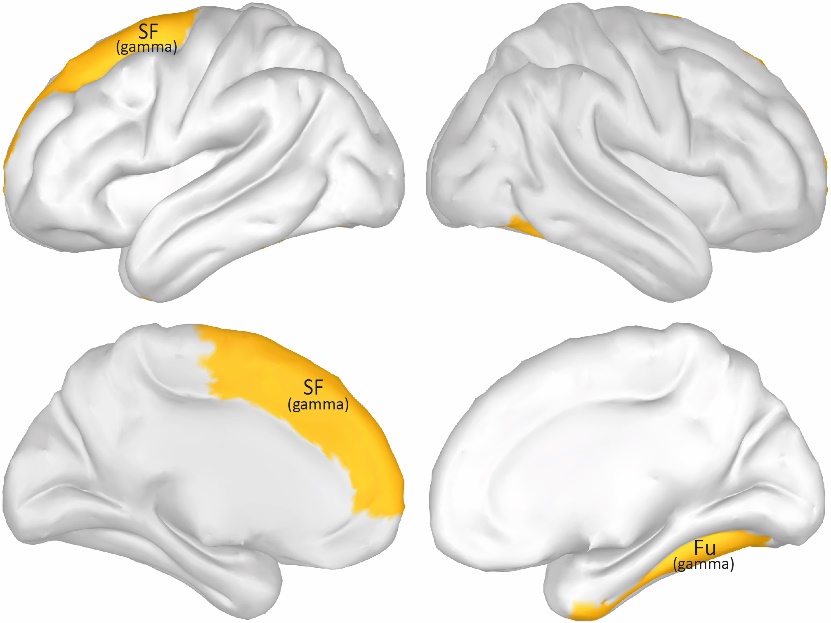


**Supplementary Figure 19. Outward Flow of Information**

**Considerations for Hearing Loss**

Since tinnitus and hearing loss are typically related conditions, it was important to verify whether our classification of tinnitus patients and healthy controls was based on neurophysiological features related to tinnitus as opposed to those related to hearing loss. To test this, we estimated the percentage of tinnitus patients with no hearing impairment (see Supplementary Figures 20 and 21). We then employed the classifier developed to distinguish tinnitus patients and controls using the entire control population and only the patients without hearing loss as the test set.

**
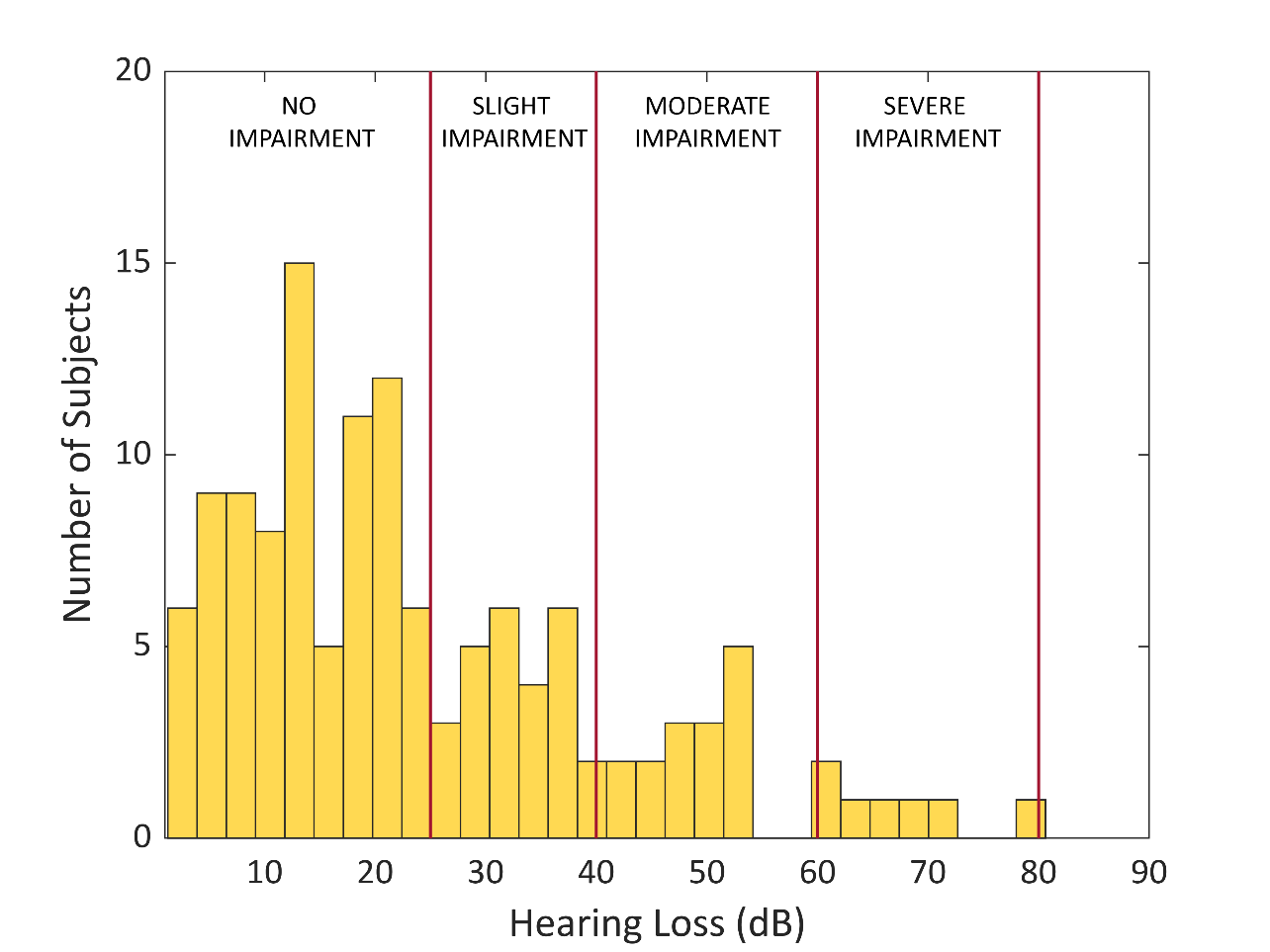
**

**Supplementary Figure 20.** Histogram reporting hearing loss distribution among the cohort of tinnitus patients. Hearing loss intervals are: i) less than 25 dB for **no impairment**, ii) 26 – 40 dB for **slight impairment**, iii) 41 – 60 dB for **moderate impairment**, iv) 61 - 80 dB for **severe impairment**, and v) higher than 81 dB for **profound impairment including deafness.** Among the tinnitus group, 61% of patients had a hearing loss lower than 25 dB, which corresponds to no impairment according to thresholds defined by World Health Organisation - Grades of hearing impairment (WHO, 2008). In accordance with these guidelines, this means that the majority of tinnitus patients had no hearing impairment. Another 22% had a mild impairment, meaning that 83% of the patients had no or mild hearing impairment.


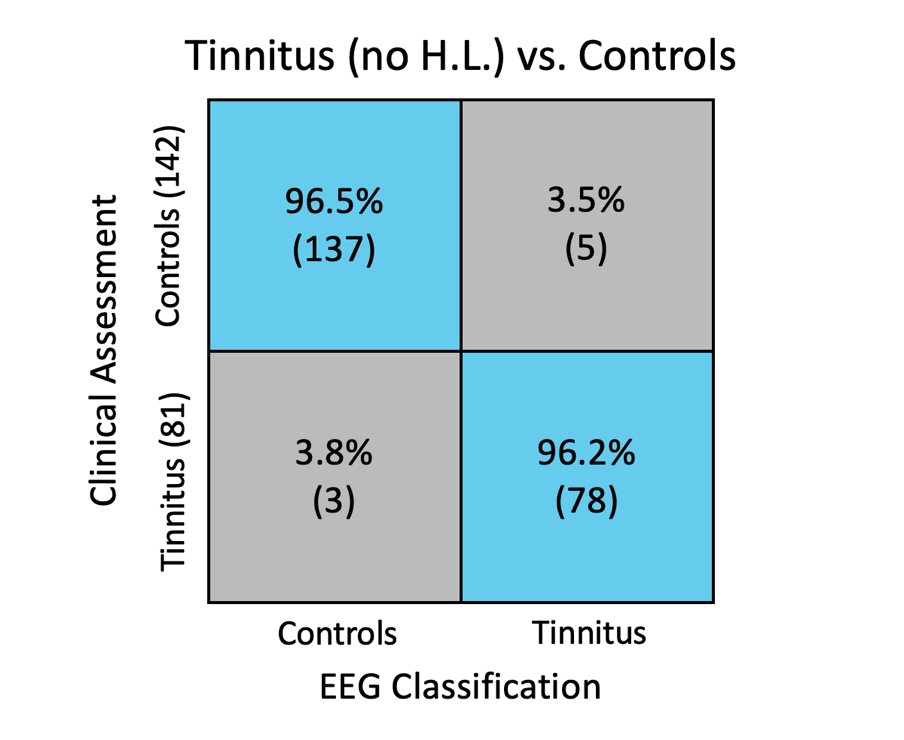


**Supplementary Figure 21.** The confusion matrix for the classification of tinnitus patients without hearing loss versus controls is presented. The clinical assessment outcomes are shown on the y-axis (with the total number of subjects written in brackets), while the predictions based on EEG features are shown on the x-axis. The diagonal elements (blue boxes) of each matrix identify the percentages of correct classifications (true positive and negative rates) for each category (the corresponding number of subjects is reported between brackets), while the off-diagonal elements (grey) identify the percentages of misclassified subjects (false positives and negatives rates). For each entry, the corresponding number of subjects that fell into the classification is shown in brackets. No H.L. stands for no hearing loss.

As apparent from the figure, the selected features perform accurately in classifying tinnitus patients without hearing impairments. 96.5% of the healthy controls (137 out of 142) and the 96.2% of the tinnitus patients with no hearing impairment (78 out of 81) were correctly classified, reaching an overall classification accuracy of 96.4%.

Taken together, these findings confirm that the selected EEG features are related to tinnitus neurophysiopathological features rather than hearing loss ones, as: i) the majority of patients, (61%) had no hearing impairment and another 22% a slight impairment; ii) the SVM classifier aimed at correctly classifying tinnitus patients **without any** hearing impairment (81 subjects, below 25 dB threshold) from healthy controls reached an overall classification accuracy of 96.4% misclassifying only 3 patients out of 81 and 5 healthy controls out of 142.

**Supplementary Appendix C: High vs. Low Distress Classification**

**Dataset Selection for PSD**

**
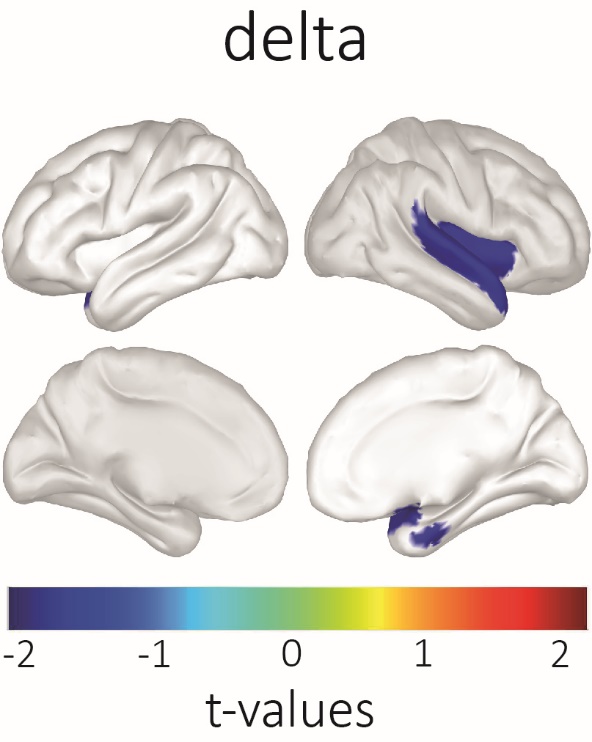
**

**Supplementary Figure 22. Band-wise PSD comparisons.** Cortical maps with regional t-values are given for the delta band, which was the only one where a significant difference was detected between high and low distress patients. Regions not showing significant differences are left uncoloured. Between-group comparisons (high vs. low distress tinnitus patients) for each band and area are conducted using unpaired t-tests.

**Feature Selection for PSD**


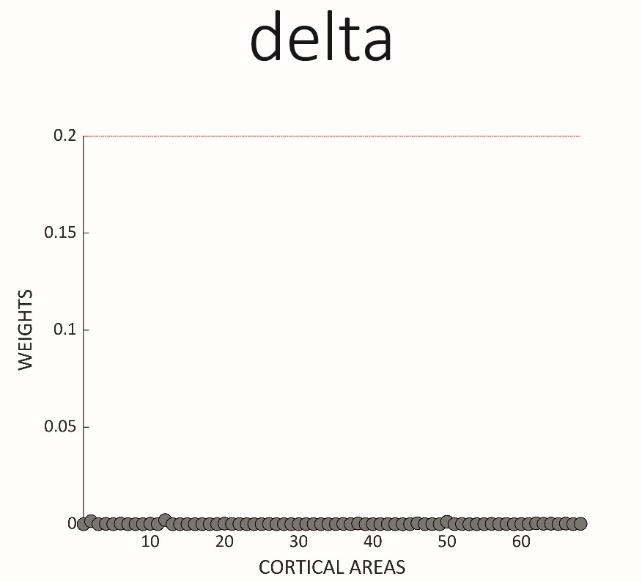


**Supplementary Figure 23. Band-wise PSD features:** For each band retained in the relative PSD feature, a scatterplot is presented to depict weights for the dataset’s cortical areas. The dashed red line represents the feature’s selection threshold. No area had a weight exceeding the threshold, and consequently no classifier feature was retained for PSD in the delta band.

**Dataset Selection for Relative PSD**


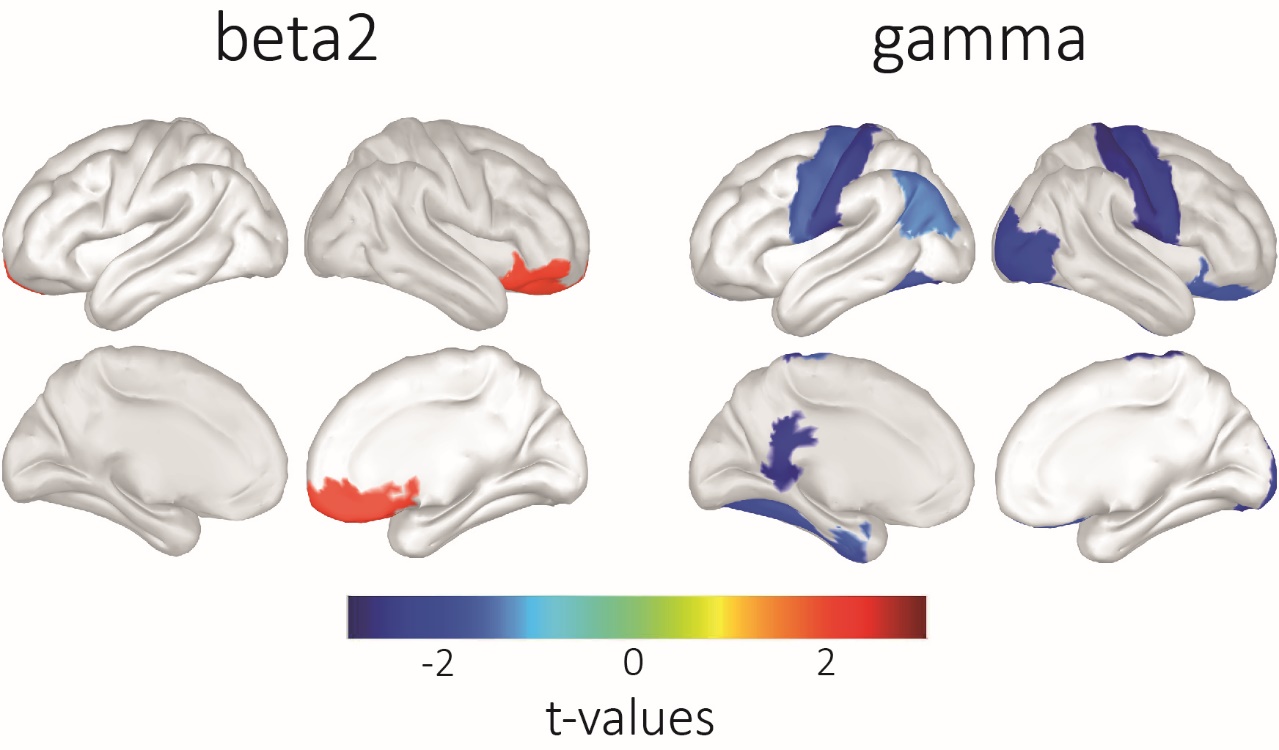


**Supplementary Figure 24. Band-wise relative PSD comparison:** Cortical maps with regional t-values are given for the bands that had at least one area showing a significant difference between tinnitus patients and controls. Regions not showing significant differences are left uncoloured. Between-group comparisons (high vs. low distress tinnitus patients) for each band and area are conducted using unpaired t-tests.

**Feature Selection for Relative PSD**


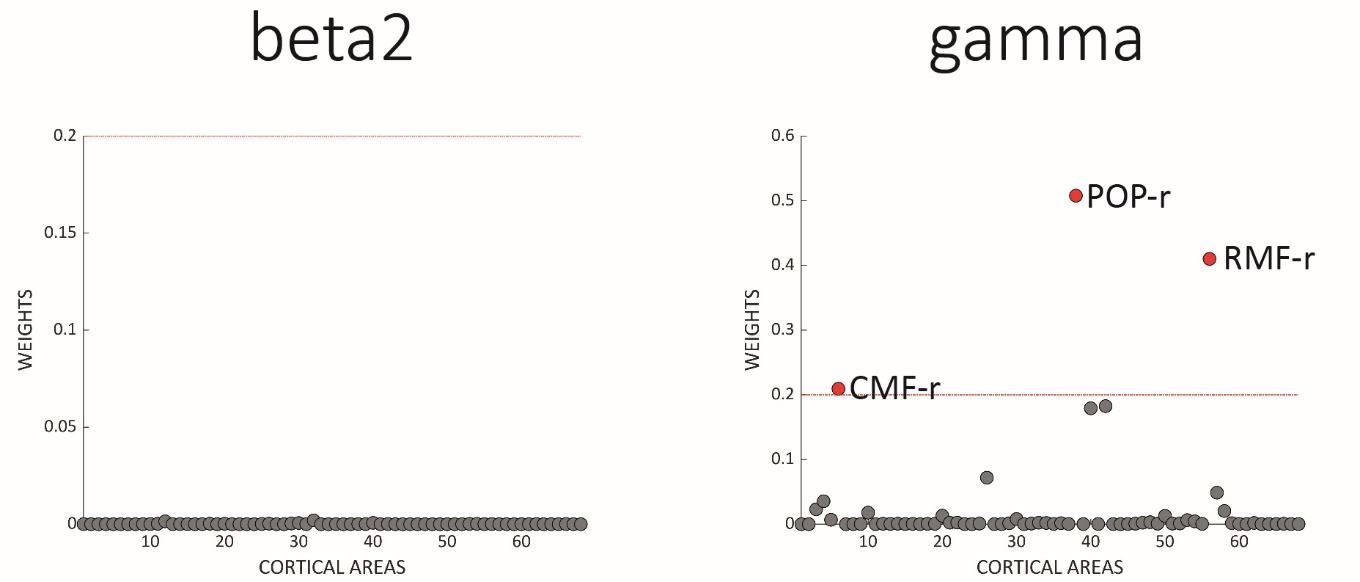


**Supplementary Figure 25. Band-wise relative PSD features:** For each band retained in the relative PSD feature, a scatterplot is presented to depict weights for the dataset’s cortical areas. The dashed red line represents the feature’s selection threshold, while red dots indicate the areas in which the feature satisfies the threshold. The red dots are also accompanied by the acronyms of their corresponding areas.

**Dataset Selection for LZC**

**
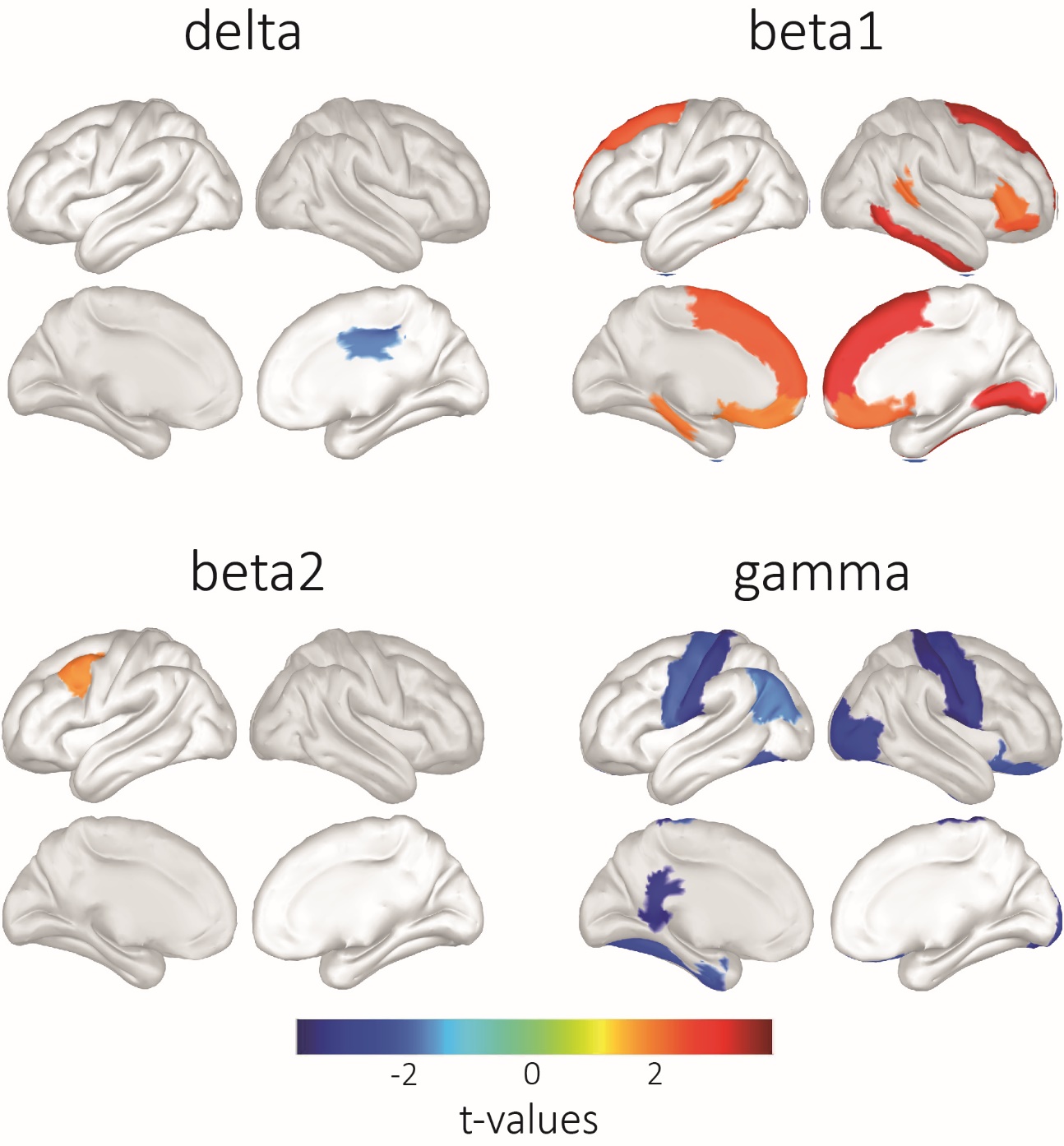
**

**Supplementary Figure 26. Band-wise LZC comparison:** Cortical maps with regional t-values are given for the bands that had at least one area showing a significant difference between tinnitus patients and controls. Regions not showing significant differences are left uncoloured. Between-group comparisons (high vs. low distress tinnitus patients) for each band and area are conducted using unpaired t-tests.

**Feature Selection for LZC**


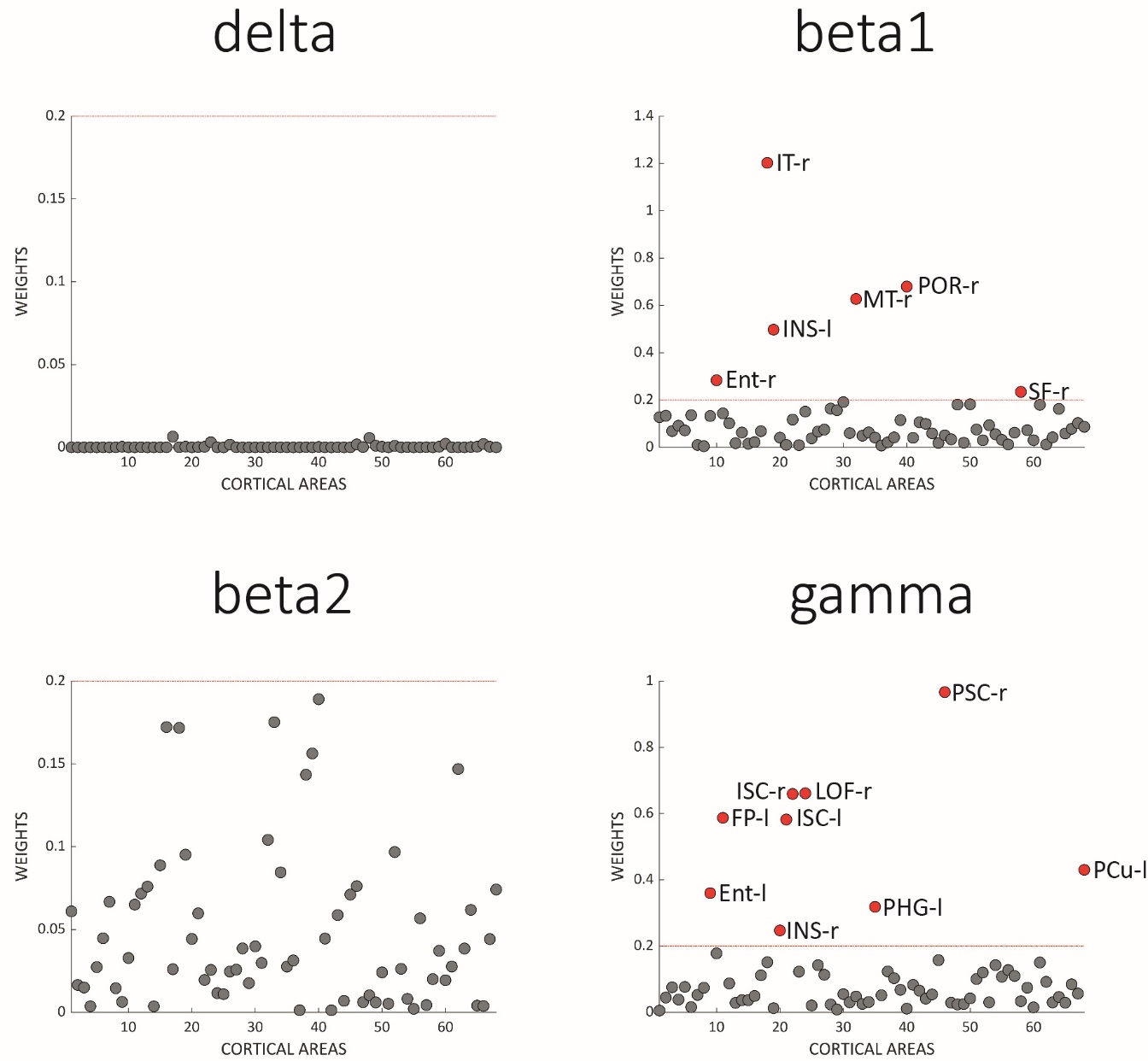


**Supplementary Figure 27. Band-wise LZC features:** For each band retained in the LZC feature, a scatterplot is presented to depict weights for the dataset’s cortical areas. The dashed red line represents the feature’s selection threshold, while red dots indicate the areas in which the feature satisfies the threshold. The red dots are also accompanied by the acronyms of their corresponding areas.

**Dataset Selection for Inward Flow of Information**


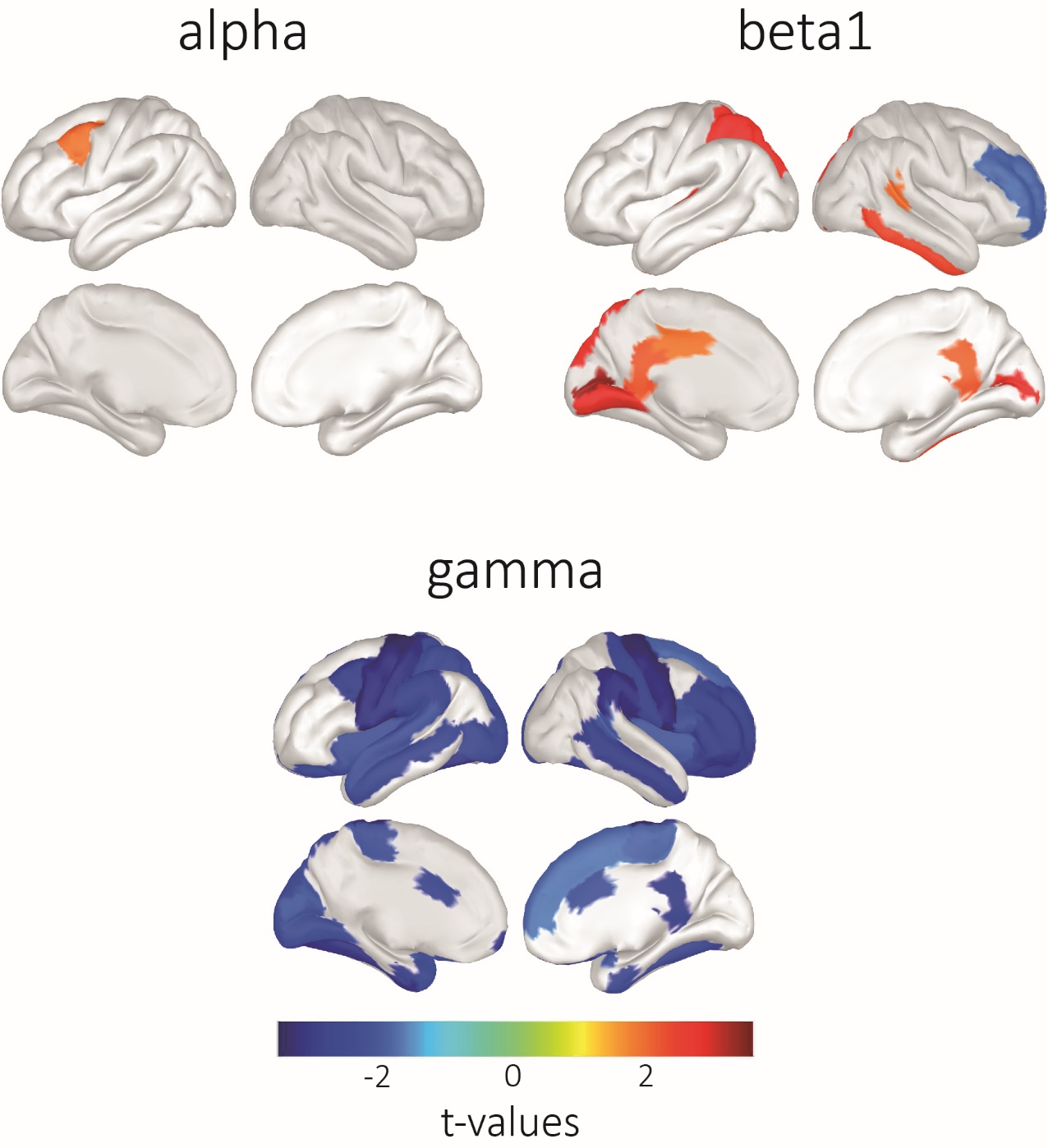


**Supplementary Figure 28. Band-wise inward flow of information comparison:** Cortical maps with regional t-values are given for the bands that had at least one area showing a significant difference between tinnitus patients and controls. Regions not showing significant differences are left uncoloured. Between-group comparisons (high vs. low distress tinnitus patients) for each band and area are conducted using unpaired t-tests.

**Feature Selection for Inward Flow of Information**

**
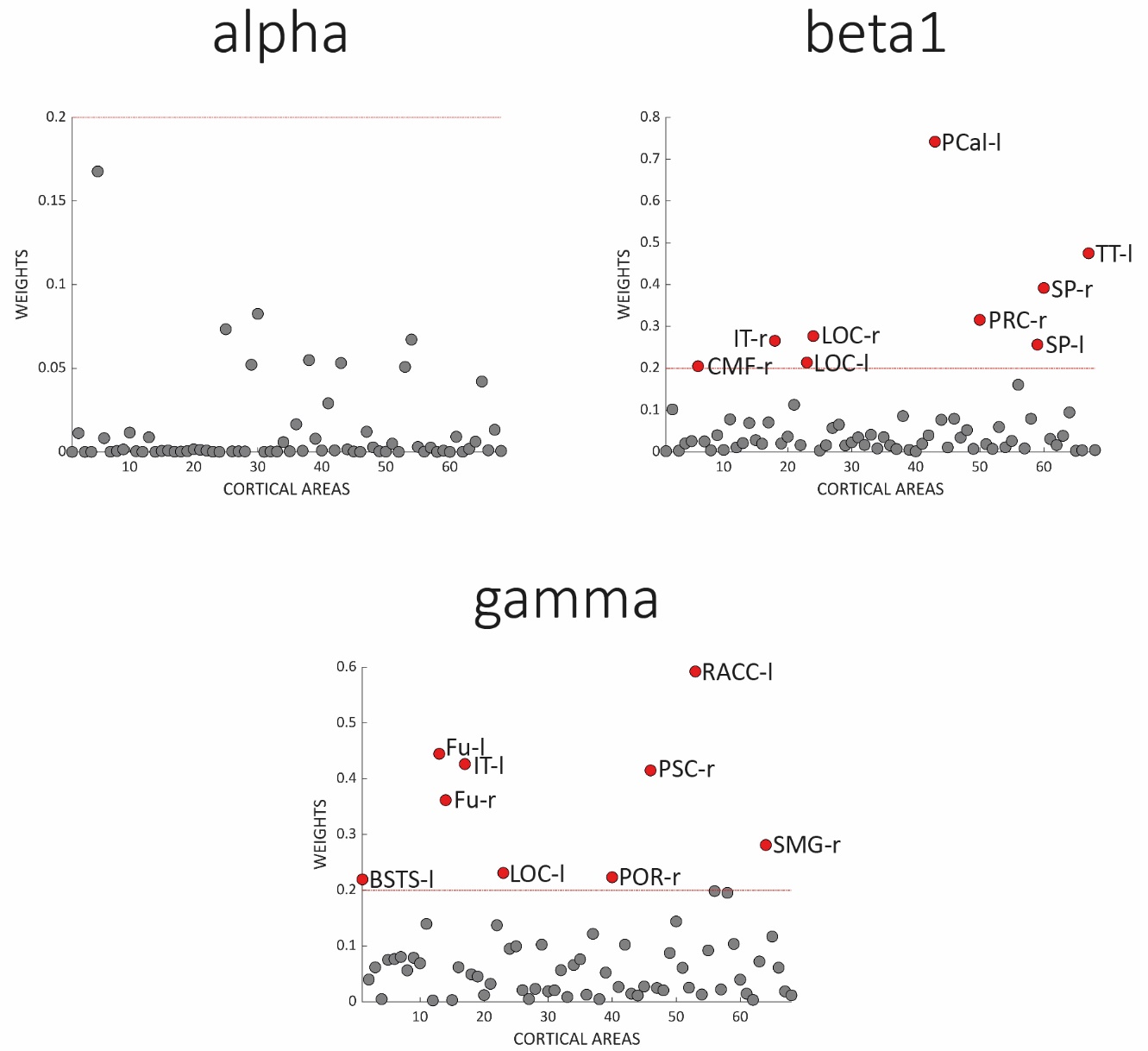
**

**Supplementary Figure 29. Band-wise inward flow of information features:** For each band retained in the inward flow of information feature, a scatterplot is presented to depict weights for the dataset’s cortical areas. The dashed red line represents the feature’s selection threshold, while red dots indicate the areas in which the feature satisfies the threshold. The red dots are also accompanied by the acronyms of their corresponding areas.

**Dataset Selection for Outward Flow of Information**

**
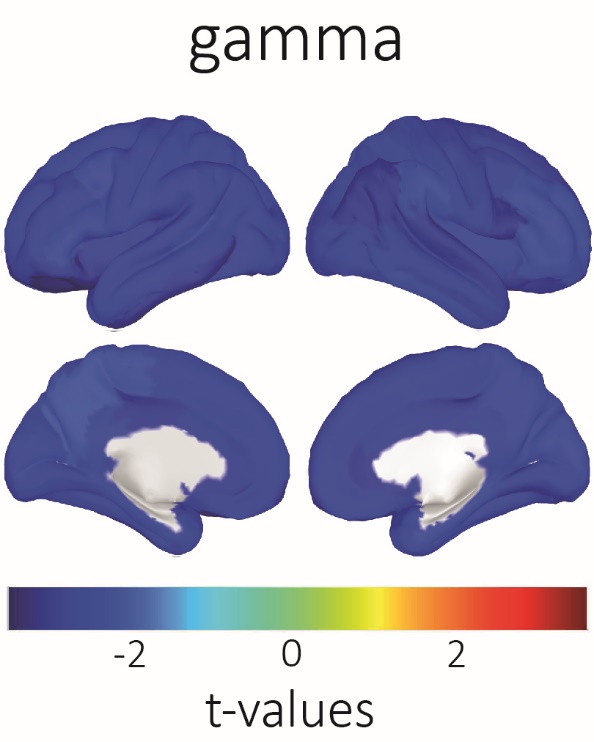
**

**Supplementary Figure 30. Outward Flow of Information comparison:** Cortical maps with regional t-values are given for the gamma band, which was the only one where a significant difference was detected between high and low distress patients. Regions not showing significant differences are left uncoloured. Between-group comparisons (high vs. low distress tinnitus patients) for each band and area are conducted using unpaired t-tests.

**Feature Selection for Outward Flow of Information**


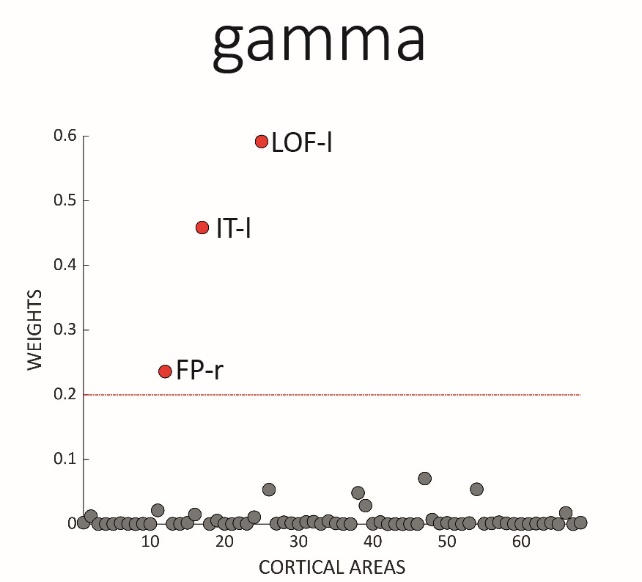


**Supplementary Figure 31. Outward flow of information features:** For each band retained in the outward flow of information feature, a scatterplot is presented to depict weights for the dataset’s cortical areas. The dashed red line represents the feature’s selection threshold, while red dots indicate the areas in which the feature satisfies the threshold. The red dots are also accompanied by the acronyms of their corresponding areas.

**Graph metrics dataset selection**

**Supplementary Table 5.** For each band and graph theory metric, the t-value and corresponding p-value related to high and low distress comparisons are reported (comparisons’ significance are assessed using unpaired t-tests). The statistics of features showing significant between-group differences are written in bold italics. (From here onwards, CC stands for Clustering Coefficient, GE for Global Efficiency, NS for Network Strength, MOD for Modularity, ID-PC for In-Degree Participation Coefficient and OD-PC for Out-Degree Participation Coefficient).

|  | **CC** | | **GE** | | **NS** | | **MOD** | | **ID-PC** | | **OD-PC** | |
| --- | --- | --- | --- | --- | --- | --- | --- | --- | --- | --- | --- | --- |
|  | t-val | p-val | t-val | p-val | t-val | p-val | t-val | p-val | t-val | p-val | t-val | p-val |
| **delta** | 1.21 | 0.23 | 0.88 | 0.39 | 1.02 | 0.31 | 1.12 | 0.27 | -0.76 | 0.45 | -1.69 | 0.10 |
| **theta** | -1.00 | 0.33 | 0.19 | 0.86 | -0.27 | 0.79 | 1.04 | 0.31 | 0.53 | 0.60 | -0.10 | 0.93 |
| **alpha** | 0.84 | 0.41 | -0.12 | 0.91 | 0.60 | 0.55 | -0.25 | 0.81 | -0.08 | 0.94 | -0.42 | 0.68 |
| **beta1** | ***2.53*** | ***0.02*** | 1.08 | 0.28 | 1.51 | 0.14 | 0.55 | 0.59 | -0.61 | 0.55 | 1.04 | 0.31 |
| **beta2** | -1.36 | 0.18 | -1.06 | 0.30 | -1.08 | 0.29 | 0.76 | 0.45 | 0.13 | 0.90 | 0.48 | 0.64 |
| **gamma** | -0.24 | 0.82 | ***-3.34*** | ***0.002*** | ***-2.76*** | ***0.007*** | 0.69 | 0.49 | -1.11 | 0.27 | -1.02 | 0.32 |

**Graph metrics features selection**


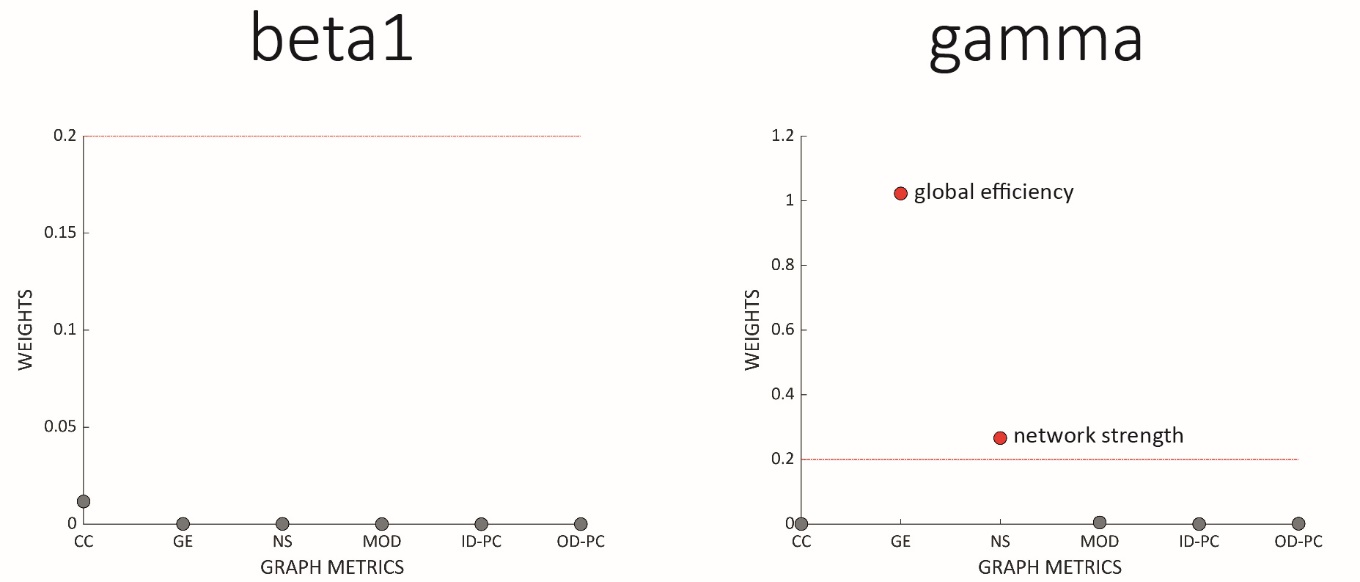


**Supplementary Figure 32.** For each retained band, a scatterplot is presented to depict weights for all graph theory metrics. The dashed red line represents the feature’s selection threshold, while red dots indicate the metrics that are above the threshold.

**Features pruning and optimization.**

**
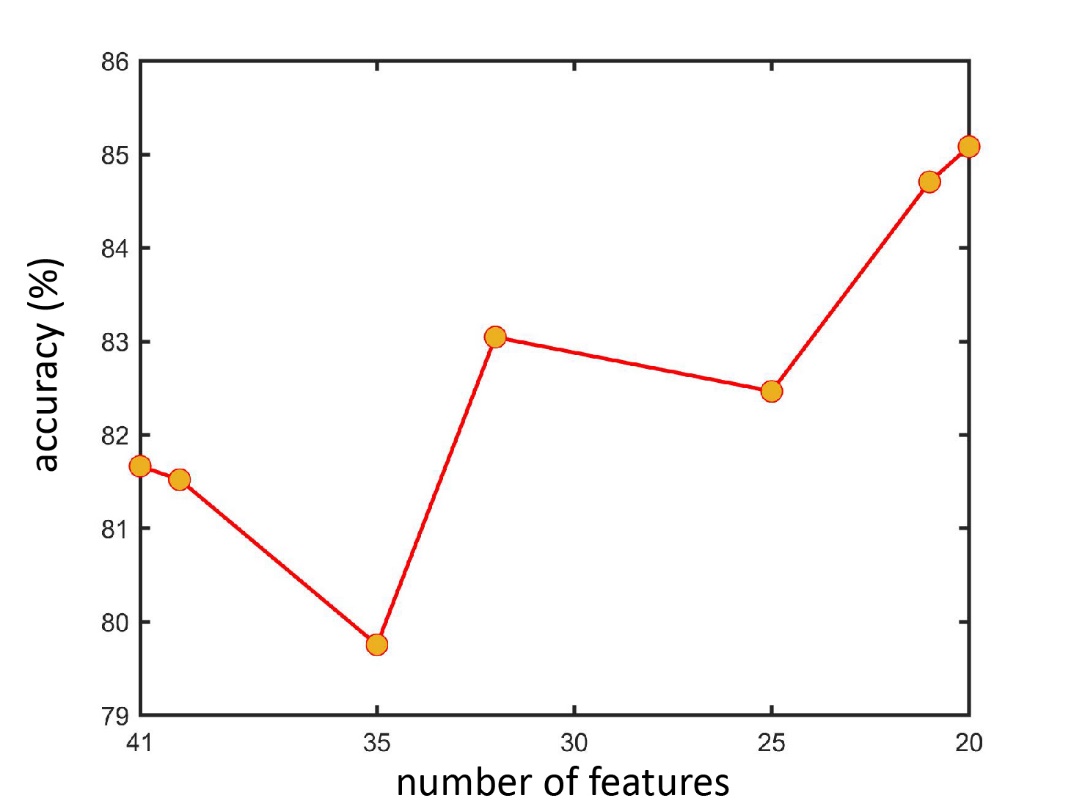
**

**Supplementary Figure 33.** The graph above shows the accuracy of the classifier versus the number of features used for it, which is decreasing from left to right in accordance with the pruning procedure.

**Final Features in High vs. Low Distress Classifier**

At the end of the features pruning and optimization procedure, which started with 41 features and a classification accuracy of 81.7%, we retained 20 features and reached an accuracy of 85.1%. In the figures below, we show the features retained for each dataset (PSD, relative PSD, LZC, Inward Flow of Information, and Outward Flow of Information) after the optimization procedure. On each cortical map, retained areas are identified in yellow. Each area is identified by its acronym and the frequency band(s) involved are specified in brackets.

Note that two graph theory features were also retained (gamma networks global efficiency and gamma network strength), though they are not shown here as they correspond to the entire network as opposed to specific regions.


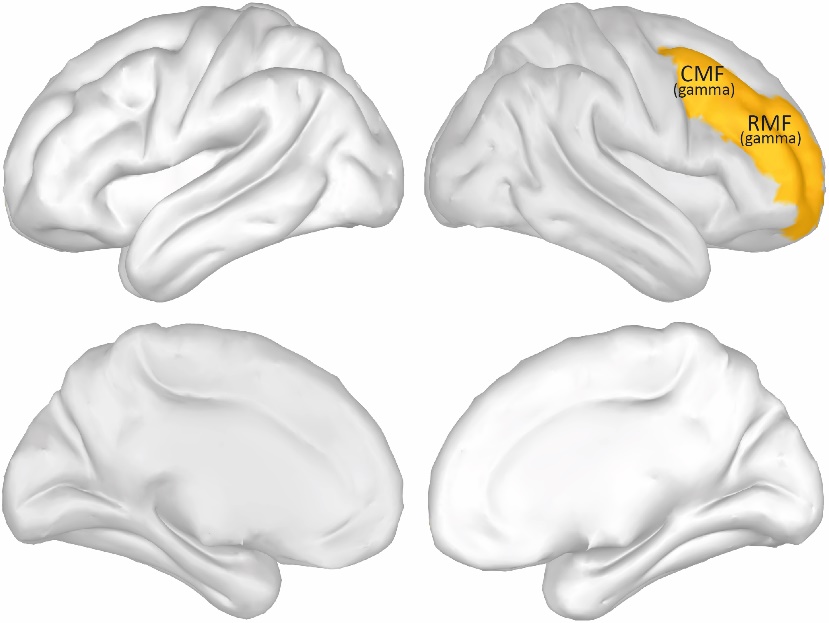


**Supplementary Figure 34. Relative PSD**

**
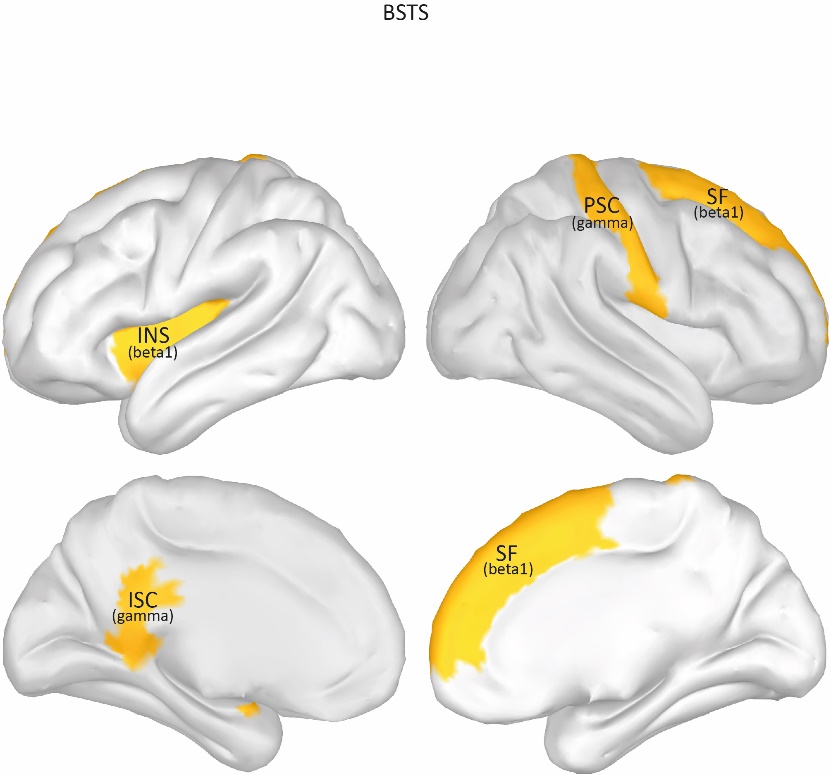
**

**Supplementary Figure 35. LZC**


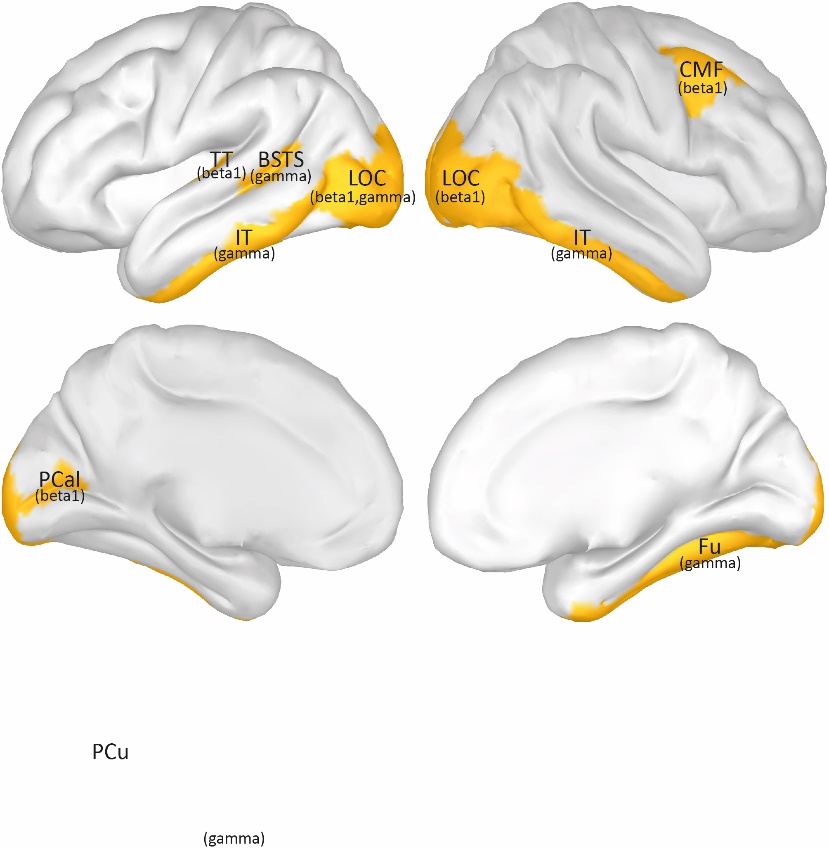


**Supplementary Figure 36. Inward Flow of Information**

**
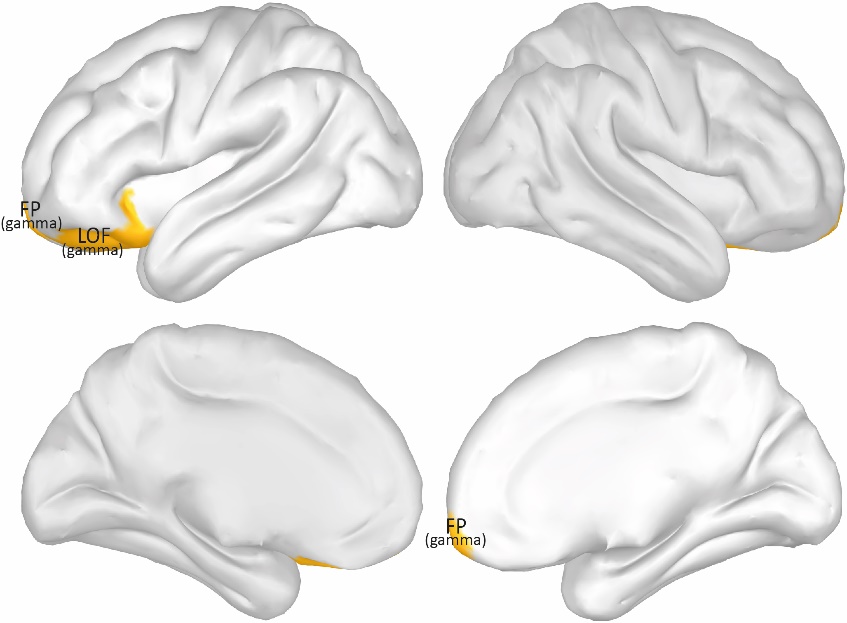
**

**Supplementary Figure 37. Outward Flow of Information**

**Demographic and Behavioural Characteristics**

Once the optimized dataset was obtained, we attempted to include all possible combinations of demographic and behavioural features in the dataset, which were then tested for their effect on the accuracy of the classifier (see Supplementary Appendix A for details on these characteristics):

- Age
- Hearing Loss
- Hearing Loss at Tinnitus frequency
- Time since Tinnitus onset
- VAS

As before, after standardizing the added features (z-score), the effectiveness of each newly generated dataset was verified by submitting it to a linear SVM with 5-folds cross-validation. The procedure was run 1000 times, and at each run we determined the classifier’s accuracy. The classifier’s overall accuracy was estimated by averaging over the 1000 runs. Results are presented in the table below.

| **Number of**  **Added Features** | **Age** | **Hearing Loss** | **Hearing Loss at**  **Tinnitus frequency** | **Time from**  **tinnitus onset** | **VAS** | **Accuracy (%)** |
| --- | --- | --- | --- | --- | --- | --- |
| 1 |  |  |  |  |  | 84.1 |
|  |  |  |  |  |  | 83.0 |
|  |  |  |  |  |  | 82.4 |
|  |  |  |  |  |  | 81.7 |
|  |  |  |  |  |  | **85.1** |
| 2 |  |  |  |  |  | 82.9 |
|  |  |  |  |  |  | 82.2 |
|  |  |  |  |  |  | 81.2 |
|  |  |  |  |  |  | 85.0 |
|  |  |  |  |  |  | 84.4 |
|  |  |  |  |  |  | 83.9 |
|  |  |  |  |  |  | 83.5 |
|  |  |  |  |  |  | 83.4 |
|  |  |  |  |  |  | 83.0 |
|  |  |  |  |  |  | 82.9 |
| 3 |  |  |  |  |  | 82.3 |
|  |  |  |  |  |  | 80.8 |
|  |  |  |  |  |  | 84.5 |
|  |  |  |  |  |  | 83.9 |
|  |  |  |  |  |  | 83.6 |
|  |  |  |  |  |  | 83.3 |
|  |  |  |  |  |  | 83.0 |
|  |  |  |  |  |  | 82.7 |
|  |  |  |  |  |  | 82.4 |
|  |  |  |  |  |  | 82.3 |
| 4 |  |  |  |  |  | 81.6 |
|  |  |  |  |  |  | 84.5 |
|  |  |  |  |  |  | 83.7 |
|  |  |  |  |  |  | 83.3 |
|  |  |  |  |  |  | 82.9 |
| 5 |  |  |  |  |  | 85.0 |

**Supplementary Table 6.** For each combination of the selected features added to the optimized dataset the classifier accuracy is reported in the last column. For each combination, the cells corresponding to the added features are highlighted in yellow. Combination obtaining the best performances are written in bold letters.

**References**

1. Jung T-P, Makeig S, Bell AJ, Sejnowski TJ. Independent Component Analysis of Electroencephalographic and Event-Related Potential Data. In: Poon PWF, Brugge JF, editors. *Central Auditory Processing and Neural Modeling*. Boston, MA: Springer US; 1998. p. 189–97.
2. Gramfort A, Papadopoulo T, Olivi E, Clerc M. OpenMEEG: opensource software for quasistatic bioelectromagnetics. *BioMed Eng OnLine*. 2010;9(1):45.
3. Pascual-Marqui RD. Standardized low-resolution brain electromagnetic tomography (sLORETA): technical details. *Methods Find Exp Clin Pharmacol*. 2002;24 Suppl D:5–12.
4. Tadel F, Baillet S, Mosher JC, Pantazis D, Leahy RM. Brainstorm: A User-Friendly Application for MEG/EEG Analysis. *Computational Intelligence and Neuroscience*. 2011;2011:1–13.
5. Desikan RS, Ségonne F, Fischl B, Quinn BT, Dickerson BC, Blacker D, et al. An automated labeling system for subdividing the human cerebral cortex on MRI scans into gyral based regions of interest. *NeuroImage*. 2006 Jul;31(3):968–80.
6. Lobier M, Siebenhühner F, Palva S, Palva JM. Phase transfer entropy: A novel phase-based measure for directed connectivity in networks coupled by oscillatory interactions. *NeuroImage*. 2014 Jan;85:853–72.
7. Chennu S, Annen J, Wannez S, Thibaut A, Chatelle C, Cassol H, et al. Brain networks predict metabolism, diagnosis and prognosis at the bedside in disorders of consciousness. *Brain*. 2017 Aug 1;140(8):2120–32.
8. Rubinov M, Sporns O. Complex network measures of brain connectivity: Uses and interpretations. *NeuroImage*. 2010 Sep;52(3):1059–69.
